# Supplementary material for: Quantitative characterization of recombinase-based digitizer circuits enables predictable amplification of biological signals
Source: Commun Biol. 2021 Jul 15;4:875. doi: 10.1038/s42003-021-02325-5 (PMC8282836; doi:10.1038/s42003-021-02325-5)
Supplement: Supplementary file 2 — Supplementary Information [file 42003_2021_2325_MOESM2_ESM.pdf]

Supplemental Information

| Supplementary Note 1: Testing Platform - Plasmid Copy Number

Supplementary Note 1.1: Approximating the Initial Distribution of Relative Plasmid Copy Number

Our testing platform (*i.e.* polyethylenimine, PEI, based transient transfection) provides a rapid test of DNA-based parts and is a likely first step in biological device development useful for circuit construction and validation. However, to incorporate the influence this testing platform has on our device performance, we considered that while transient transfection comes with the advantage of testing varying levels of circuit copy number in a single experiment, these copy numbers will dilute out of the cell given sufficient time and that there exists an initial delay from the time of transfection to time the transfected DNA can make it to the nucleus for transcription. We evaluated each of these areas to model the effect of working with transient transfection data. As previous work also considered these areas and was able to make useful predictions, we focused our modeling efforts in these areas ([1, 2, 3]).

Transient transfection comes with the advantage of testing varying levels and different combinations of plasmid copy numbers in a single experiment. However, to describe the different plasmid combinations present in each cell we need a method to parse the data by approximate circuit copy numbers. There is one transcriptional unit, TU (i.e.promoter/gene/terminator), per plasmid and the relative copy number between the transcriptional units is varied by varying the weight ratio of plasmids used in the transfection. Therefore, we know the global ratio between plasmid used in each experiment, but we must consider that some cells will receive slightly disparate ratios of the plasmids or total plasmid content regardless of the intended ratio or weight. This variation can be approximated as we will describe later and is shown in Fig. S9. We refer to this variation as the initial transient distribution in plasmid copy number.

Our circuits are comprised of upto 6 TUs: CAG-mRuby, CAG-rtTA, TRE-BFP, TRE-Flp, FRT-GFP and (U6-shRNA or U6tetO-shRNA). However, we can simplify the dimensionality of the initial transient distribution space from which our data is derived by taking note of the following relationships with the following assumptions: 1) The distribution including mean and covariance of CAG-mRuby should equal that of all the other TU's including FRT-GFP. This variance within a single transfection will also vary less compared to independent transfections of these plasmids, given the number of plasmids used in the transfection. 2) The variation between the copy number of two plasmids within a single cell is much less than the variation of plasmid copy number across cells. Fig. S9 highlights these relationships 1) and 2) and is a pool of three biological replicates of the experiment each containing three technical replicates each.

Given a relationship between fluorescence expression and plasmid copy number, we chose to use the distribution of constitutive fluorescent protein (CFP), ((*e.g.*) mRuby expression) to approximate the plasmid distribution of plasmids in the system. Additionally with TRE-BFP fluorescence expression, we can also approximate TRE-Flp copy number. However, the relationship between BFP and Flp is also affected by the expression of shRNA in the system. Assuming the governing source of variation is between cells and not within a single cell, when no shRNA is present we can derive a relationship between BFP expression and Flp expression given a relationship between fluorescence expression and plasmid copy number. When shRNA is present we will need to further take into account the topology of the circuit as modeled in supplementary note Chemical Reactions. Altogether this then shows promise of giving us a method for which to define inputs to our circuit in terms of fluorescence and outputs of a digital enhancer in terms of fluorescence while considering the variation introduced by the testing platform.

* Equally contributing authors.

Supplementary Note 1.2: Modeling the Color Control: relationship between fluorescence expression and plasmid copy number

To define a relationship between fluorescence expression and plasmid copy number we modeled the expression of a color control sample over time. This color control consists of three TUs at a 1:1:1 weight ratio and non-expressing filler DNA.As our transfection is mediated by the formation of PEI DNA complexes, it appears there must be a variation in the size of the many complexes formed during the transfection where the larger complexes have larger plasmid numbers but the same relative ratio of plasmids compared to the smaller complexes [4]. The variation in proposed complex size appears to be the governing source of variation as seen in Fig. S9. Our color control used 31.25ng of CAG-BFP, 31.25ng of CAG-GFP, 31.25ng of CAG-mRuby and 156.25ng of filler DNA to get a 1:1:1 ratio of TUs containing a single fluorescent protein in each. We then measured the fluorescence value of this sample overtime via flow cytometry. We repeated this experiment three times and pooled all the data for creation of the histograms. The variation between technical and biological replicates is further investigated in fitting the initial delay and plasmid dilution as described in supplementary note Plasmid Dilution. We can see in Fig. S9 for a given time point that there is a linear trend between the TU outputs whose variation is smaller than the range of expression along the axis of a single color. It is important to remember these are measures of fluorescence not measures of plasmid copy number.

To derive a method for approximating relative copy number from fluorescence, We can get a relative measure of plasmid copy number for a constitutively expressed fluorescent protein, such as CAG-mRuby, by solving the discrete birth death process described in equation (1). By noting the repeating behavior in this discrete system as time progresses we can come to equation (2). Therefore given a fluorescence value, time of collection, Molecule to MEFL, birth rate, death rate and plasmid division time we can approximate the plasmid copy number. However we do not know plasmid division time, Molecule to MEFL, birth or death rate. Although we will fit the data for plasmid division time as described in supplementary note Plasmid Division, considering the other constants we can only approximate a number for plasmid copy number that is monotonic, however possibly non-linear, in relation to degradation rate. We will call this number relative plasmid copy number, RCN. Due to the non-linear relationship between this number and the death rate we choose a biologically relevant number for degradation rate and assumed a production and Molecule to MEFL rate of 1. The chosen biologically relevant death rate was chosen to be $0.02/\text{hr}$ as this is a central approximation seen in a survey of protein decay rates from the literature [5]. As we construct the model we will see that the predictive power of the model is unaffected by the values we choose for Molecule to MEFL, birth and death rate and only affects the absolute value of the fit parameters and the error of approximating these numbers. This is expected due to both the monotonic relationship of the relative plasmid copy number to Molecule to MEFL, birth and death rate as well as linearly proportional relationship relative copy number has will the parameters in the model it is multiplied with.

As a proof of concept and to highlight the need of adding an initial delay and/or plasmid dilution to our model, we simulated the time progression of our color control sample with the ODEs found in the equation set (3), where RCN_{mRuby}, RCN_{BFP} and RCN_{GFP} were estimated from the 96 hour time point. b and d_p were set to $1/\text{hr}$ and $0.02/\text{hr}$ respectively. 1000 cells were simulated and we compared the results of these simulations to the experimental data (Fig. S10). As solved for we capture the system behavior at the 96hr time point, however, we are missing the delay in expression at the 6hr time point. This experimentally observed delay is expected as it is thought to take some amount of time for the transfection complexes to reach the nucleus, and further time for transcription and translation to occur. However, as we will see in the next section plasmid dilution does not appear pertinent to being able to further describe the observable trends in the data beyond what the current modeled reactions already give us. One more concept we would like to highlight in this section before moving onto plasmid dilution, is we will need to also add the initial delay and plasmid dilution to our estimate of relative plasmid copy number (4). This formalization is

very similar to our previous work [1, 2] and described in more detail in the supplementary note Plasmid Dilution.

Supplementary Note 1.3: Adapting This Method to for a Minimal Model of shRNA Regulated Recombinases

To adapt this approach to for our minimal model of shRNA regulated recombinases, we further assumed we can use the fluorescent markers in an experiment to approximate the multi-dimensional distribution of the TU's used in the samples by re-centering the distribution assuming linear proportionality between the ratio of ng used in the sample to those used in the plasmids constitutively producing fluorescent proteins. (*e.g.* If the sample TU ratio is 1:10ng between a TU not constitutively expressing a fluorescent protein and a TU that is constitutively producing fluorescent proteins we would divide the RCN of the fluorescent TU by 10 to approximate the distribution of the non-fluorescent TU RCN. With this in mind, to approximate RCN for the two assumed key TUs in our system FRT-GFP and TRE-Flp, we used the corresponding fluorescent values as follow: Constitutive expressed CAG-mRuby was used to approximate the reporter plasmid, FRT-GFP, by taking into account the difference in ng used between these two TUs as described above. To use TRE-BFP to approximate RCN for the TRE-Flp expression is harder as it is also influenced by the shRNA production in the system. For these we chose to derive a map from RCN calculated from our TRE-BFP to TRE-Flp expression while taking into account the topology of the system as described in Supplementary Note Chemical Reactions. Also the other TUs are addressed individually in the Supplementary Note Chemical Reactions when making the map from BFP to Flp.

$$\begin{cases} \frac{CFP(t+\Delta t)}{M2MEFL} = b * pcn * \phi(t) + (1-d) * \frac{CFP(t)}{M2MEFL} \\ \phi(t) = 0.5^{\lfloor \frac{t}{\lambda} \rfloor}, \end{cases} \tag{1}$$

where $CFP(t)$ represents the constitutive fluorescent value coming from CAG-mRuby at time point t. b and d stand for protein production and decay rates. pcn stands for plasmid copy number. $M2MEFL$ is then the Molecule to MEFL proportionality constant. λ is cell division time. $\phi(t)$ describes decreasing plasmid copy number due to cell division.

$$RCN = \frac{FP(t)}{\sum_{i=1}^{t} \phi(i) * (1 - d - \log(2)/\lambda)^{t-i}}, \tag{2}$$

where RCN stands for relative plasmid copy number. $FP(t)$ represents the florescent value in arbitrary units, A.U., coming from CAG-mRuby and time point t. d stands for the decay rate and is set to 0.02/hr as an average value of protein degradation as reported in this referenced survey [5]. λ is cell division time.

$$\begin{cases} \frac{dP_{mRuby}}{dt} = bRCN_{mRuby} - dP_{mRuby} \\ \frac{dP_{BFP}}{dt} = bRCN_{mRuby} - dP_{BFP} \\ \frac{dP_{GFP}}{dt} = bRCN_{mRuby} - dP_{GFP}, \end{cases} \tag{3}$$

where P stands for proteins. Subscripts "mRuby", "BFP" and "GFP" represent their respective florescent proteins. b and d are protein production and decay rates, respectively.

Considering delay in gene expression, we rewrite RCN as:

$$RCN = \frac{FP}{\sum_{i=t_i d}^{t} \phi(i) * (1 - d - \log(2)/\lambda)^{t-i}}, \tag{4}$$

where $t_i d$ is the initial delay time due to the gene expression delay seen in transient transfection data.

Supplementary Note 2: Testing Platform - Plasmid Dilution and Initial Delay

Supplementary Note 2.1: Plasmid Dilution and the Initial Delay

Our previous model of transient transfection takes into account both plasmid dilution due to cell division and an initial delay in the production of fluorescence [1, 2, 3]. There are many reasons to have this initial delay. Three of which are as follows: 1. Transfection complexes need to make their way from the surface of the cell to the nucleus. 2. The initial shock of the transfection reagents is hard on the cells and they may need sometime to recover before they can jump into transcription of these plasmids. 3. As we are measuring fluorescence there will be a difference in maturation time for the various fluorophores in our system (*i.e.* mRuby folds on the scale of hours whereas eGFP and eBFP fold on the scale of minutes) [6] [7]. From the experimental time course data in Fig. S11 Panel A, as expected there appears to be a delay in the initial start time for observable fluorescence for each cell that varies with which type of fluorophore that was used (*i.e.* eBFP and eGFP show appreciable signal at 6hr where mRuby needs more time). The difference between the mRuby and eBFP/eGFP fold times is much less that the size of the initial delay, considering this we assumed this difference insignificant. We can see that all the cells do not start fluorescing at the same time, therefore it is logical to conclude that there is a distribution of delay times found in a particular population. One hypothesis for the cause of this distribution is that these delay times result in plasmids only entering the nucleus upon cell division. To explore this hypothesis we assumed a uniform distribution of start times with range 0 to length of cell division time. We then used our system of ODE's to simulate a population drawing a delay time from this distribution for each cell and cutting all molecular species in half every time a multiple of the cell division time was reached. (Note: The uniform distribution was chosen as we do not expect there to be any bias in how far along our cells are in their cell cycle.) We started with a division time of 19.8hr following our previously published results [1]. (Aside: Auto-florescence was assumed to have an additive effect in the model and was captured by adding a random value drawn form the wild type sample fluorescence output to the output of each fluorescent protein in a given simulated cell after the ODE simulation was complete.) Results of our initial simulation that included plasmid dilution can be found in Fig. S(11 Panel B). We can see that this does NOT capture the initial delay behavior and seams to overshoot the time course behavior. We also tried fitting plasmid dilution to the time course data using a RMSE loss function comparing the estimated probability distributions. This improved the results by increasing the division time to near 50 hour. However we are still not capturing the initial delay (Fig. S11 Panel C).

Supplementary Note 2.2: Initial Delay and Plasmid Dilution: A Non-uniform Distribution for Initial Delay and Plausible Reason(s) for the Lack of Observable Plasmid Dilution

Considering, for initial delay, a uniform distribution whose range is the cell division time does NOT appear to capture the behavior we are seeing, the question becomes what would capture this behavior. We tried a Gaussian distribution of delay times with a mean equal to the ratio of the division time to μ_{ID} and the standard deviation equal to σ. This model was then fit to the time course data in the same manner as before. Initial value we adjusted by hand to get a near approximation of the data to improve fitting speed and chance of finding a minima. The results of this fit can be found in Fig. S12. The fit values for the division time, μ_{ID} and σ are 96, 11.5 and 8.4 respectively. The bias toward

a central value in the distribution of delay times perhaps is related to the initial shock of transfection. Also it is curious why such large division time fits our data when our previous data suggest that a division is around 19.8. Since the fit division time is essentially the length of the simulation it is suggesting we do not see any appreciable decrease in signal as a result of the cells dividing. One reason as to why this may be the case is resource competition. In other words, it is likely previous experiments seen in the [1] had lower transfection efficiency compared to our current experiments which have near 100% transfection efficiency. As the plasmid number increases in our cells we expect the ribosomes and other transcriptional resources to become the rate limiting factor [8]. Another hypothesized explanation is that too few divisions happen in 96hrs for the plasmids to be diluted enough to see a noticeable effect in the nucleus of the cell.

During this dilution analysis, we noticed that half of the plasmids used for experiments testing the digital enhancer module contain an SV40 origin of replication (ORI): BW339 (FSF-GFP output), BW363 (blank), LC20 and LC41 (shRNA plasmids), and all color controls (BW361, BW462, BW465). Mammalian cells expressing the SV40 large T antigen are able to replicate plasmid DNA containing the SV40 ORI, and all of the HEK293FT cells used in these experiments express this antigen. Therefore, we wanted to check if some of the plasmids showed different dilution behavior compared to others. To test whether the SV40 ORI was having a significant impact on the performance of our devices, we constructed a plasmid almost identical to the eGFP single positive color control (CAG-GFP-ORI+) used in all experiments, but that does not contain the SV40 ORI (CAG-GFP-ORI-). We transiently transfected the SV40 ORI+ and ORI- plasmids into separate wells of HEK293FT cells in triplicate, and collected flow cytometry data over a four day period to examine the differences in max expression of each plasmid. We note that there does not seem to be any significant difference in eGFP expression from cells transfected with either plasmid up to 96 hours post transfection, and there is no significant difference between population level eGFP expression at 48 hours (Fig. S13).Therefore we chose to move forward with no dilution or replication incorporated into the model as plasmid replication and /or dilutions should not be having a significant effect on our system as we have characterized it.

Removing cell division we refit with the initial delay mean being μ_{ID} instead of the ratio of the division time to μ_{ID}. Results of this fit are μ_{ID} and σ fit to 9.5 and 5.5 respectively giving the behavior depicted in Fig. S12A. To further support this choice in initial delay we can note that the lower mode in the experimental data is likely governed by auto-florescence of the cells and the loss of this lower mode in our experimental data takes longer than the maturation time of the fluorescent proteins. Overlaying the wild type cell auto-florescence at 96 hr for each of the channels used to collect the Multi-Color control with the 6 hour time point of the corresponding fluorophore shows this behavior (Fig. S11). This trend is also true for the other fluorescent values. (Aside: As expected, the exact amount of auto-florescence is dependent on PMT being used to collect the particular fluorescence channel.)

Supplementary Note 2.3: Variation in Multi Control Data: Error in Estimation and Comparing to Experimental Data

If we break apart the three replicates as seen in Fig. S12B we can see their is a visual difference between the replicates. Fitting initial delay μ_{ID} and σ_{ID} and plasmid dilution separately to each one of these replicates independently give use a range of values [8.0-13.6] for μ_{ID} and [3.7-14.1] for σ_{ID} with the most extreme behavior making up the set (μ_{ID} = 13.7, σ_{ID} 3.6 and μ_{ID} = 8.1, σ = 7.9). The fits reflecting these results can be found in Fig. S12D-E. As we suspect this variation to be mostly governed by the health of the cells which we do not have a direct measure of, in later sections of this paper we consider this possible range in behavior in terms of our model predictions to show this variation does not significantly impact our results in terms of fitting for leaky expression and degradation.

Supplementary Note 3: Cellular Reactions

Supplementary Note 3.1: Model Construction

We expect the most informative model will be one that balances complexity with usability; therefore our goal is to first construct the simplest model capable of explaining the data and then explore the effect of adding back in complexity. To start we surveyed the literature as described in the main text. We then worked to describe the key features of the testing platform as described in the Testing Platform supplementary note. Here we described how we used these features to build a multi-layered (phenotypic and mechanistic) model similar to our previously published work [1, 2, 3]. The mechanistic part of our model is a set of ODEs describing the purposed governing reactions. We chose to use ODEs instead of SDEs because we believe our system to reside in a large molecular number regime. The phenotypic part of our model is incorporated by feeding in the distribution of fluorescence values collected in the experiment into the model as rate constants and initial conditions. It is important to note this also incorporates the variability we see in the data under the assumption that the governing source of this variability comes from differences in plasmid copy number. Details of this assumption can be found in the Testing Platform supplementary note. Using the Law of Mass Action, LMA, to derive a set of ODEs followed by considering rate limiting reactions, separation of slow and fast time scales and the conservation of mass we formed our minimal model. To elucidate if this minimal model was sufficiently complex we built a more complex model taking into account a larger set of proposed states for comparison and found little to no clear benefit of increasing system complexity. After evaluating the performance of our model to explain the data we use this model in the main text to look for predictive trends in behavior useful for circuit design. We then tested those predictions showing that the expected qualitative trend is realizable.

Supplementary Note 3.2: Proposed Mechanistic Governing Bio-Chemical States

As mentioned in the main text we are starting by taking the three states depicted in Fig. S14. The list of chemical reactions describing the transfer between these states in the context of each topology can be found in table (1). Using LMA, for all these reactions we obtain a set of ODE describing the system behavior (Eqns. 5 - 15). Variables and Species are described in 2 and 3. Grouping these equations by slow and fast process we can see we have some equations that contain both fast and slow processes (*i.e.* mixed equations). Two of these equations can be accounted for taking note of conservation of mass and assuming $rtTA_i$ is never rate limiting due to the large amount of ng used for expressing this plasmid in each experiment. The last of these equations we can consider by mapping to a space where $Z = P_{FLP} - 4D_{GFP,i}$ (*i.e.* Eqn. 16). Scaling time by a slow rate constant, d_p and letting $\epsilon = \frac{d_p}{f_1}$, we can see as the difference between the slow rate constant and a fast rate constant, f_1, gets large $\epsilon \longrightarrow \emptyset$. Finally with a bit of algebra and remembering the conservation of mass $D_{GFP,T} = D_{GFP,i} + D_{GFP,a} + D_{GFP,c}$; $U_{6,T} = U_{6,i} + U_{6,a}$, we can arrive at (Eqns. 17-22). Mapping back to the original space we can see the complexity for $\frac{P_{FLP}}{dt}$ is a bit unruly. However, it is evident in equations 23-30 that both part2 and part3 converge to 0 as P_{FLP} gets large and the region where P_{FLP} in small we are already assuming is negligible as we are working with systems that have large molecular numbers. We would like to point out that this is in effect assuming the retroactive effect involving P_{FLP} is negligible. However it is worth noting that a similar retroactive effect will occur with the addition of some of the states we are assuming to be non-governing. We will look at the effect of adding back in sequestration of P_{FLP} simulating a possible retroactive effect to investigate this assumption further later in this supplementary note.

Supplementary Note 3.3: Phenotypic Interactions within the Testing Platform

As described in the supplementary note Testing Platform, we are measuring the inputs and outputs to the digital enhancer systems in terms of several unique fluorescence signals. Thus, we can use the distribution in of the fluores-

cent signal strength to estimate RCN for both FRT-GFP and TRE-BFP. (*i.e.* We will let $D_{GFP,T} = ngGFP \cdot r \cdot RCN_{GFP,T}$ and $D(rtTA)_{FLP} = ngFLP \cdot \alpha \cdot RCN_{FLP}$) Subsequently $D_{GFP,x} = ngGFP \cdot r \cdot RCN_{GFP,x}$ where $RCN_{GFP,x}$ is not directly calculated but rather estimated from RCN_{CFP} using the conservation law. RCN_{FLP} can, in a like manner, may be estimated from RCN_{IFP}.

As D_{FLP} is inducible, by using RCN_{IFP} at different $rtTA_a$ values we are essentially assuming rtTA$_a$ is not rate limiting. Therefore, a first order birth rate from mFlp production coming from the $D(rtTA_a)$ is assumed. Similarly, we can make the same assumption for shRNA by setting $U_{6,T} = ngshRNA \cdot \kappa \cdot RCN_{U6T}$, where RCN_{U6T} will also be estimated from RCN_{CFP}. The concentration of $rtTA_a$ in the system will vary monotonically with the amount of IFP at a given time point. As the concentration of $rtTA_i$ is already chosen to be non-rate limiting and the binding rate of DOX is already identified as fast compared to the slow reactions, we chose to model the relationship between IFP and rtTA as being linearly proportional. We realize that this relationship may not be linear; however, we chose to move forward with a linear assumption until and more complex representation appears to be justified. Considering this relationship between IFP and $rtTA_a$ we let $rtTA_a = \eta IFP$, where IFP represents a relative $rtTA_a$ amount ($RrtTA_a = IFP$) . Substituting in these relationships and renaming grouped parameters as found in our list of final parameters (table 4), we arrive at our mixed phenotypic/mechanistic model seen in equations 31 - 35.

Supplementary Note 3.4: How does making the model more complex affect the system?

After the model was parameterized, as described in the Parameterization supplementary note, we chose to explore a more complex model incorporating retroactive effects on Flp to determine how this phenomenon affects our system, such as the retroactive effect on FLP production. Does modeling this extra complexity change the behavior of the system? To look into this question we added in an additional state for the system based on $4\,\mathrm{P_{FLP}} + \mathrm{D_{GFP,a}} \xrightleftharpoons[\mathrm{r_r}]{\mathrm{f_r}} \mathrm{D_{GFP,c}}$, where f_r and r_r are forward and reverse binding rates or Flp to left over FRT sites after the excision. Fig. S15 is a representative image of the repressive effect this has on the system's induced on state. Even after refitting the parameters we failed to see a reasonably constrained amount of improvement in the fits. Furthermore, after exploring parameter space, a major difference between this more complex model and our minimal model was the ON-state's non-monotonic trend overtime in the sequestration model. However, this non-monotonic behavior was not evident in our experimental data. Therefore, we chose to move forward with the minimal model of our system.

Initial Set of ODEs

Slow Reactions

$$\frac{dP_{GFP}}{dt} = t_l m_{GFP} - d_p P_{GFP} \tag{5}$$

$$\frac{dD_{GFP,a}}{dt} = f_2 D_{GFP,c} \tag{6}$$

Mixed Reactions

$$\frac{dP_{FLP}}{dt} = t_l m_{FLP} - d_p P_{FLP} + 4f_2 D_{GFP,c} - 4f_1 D_{GFP,i} P_{FLP}^4 + 4r_1 D_{GFP,c} \tag{7}$$

$$\frac{dD_{GFP,c}}{dt} = f_1 D_{GFP,i} P_{FLP}^4 - r_1 D_{GFP,c} - f_2 D_{GFP,c} \tag{8}$$

$$\frac{drtTA_i}{dt} = p_r - d_p rtTA_i - f_4 DOX rtTA_i + r_4 rtTA_a \tag{9}$$

Fast Reactions

$$\frac{dD_{GFP,i}}{dt} = -f_1 P_{FLP}^4 D_{GFP,i} + r_1 D_{GFP,c} \tag{10}$$

$$\frac{dU_{6,a}}{dt} = -f_3 rtTA_a U_{6,a} + r_3 U_{6,i} = -\frac{dU_{6,i}}{dt} \tag{11}$$

$$\frac{dm_{GFP}}{dt} = p_1 D_{GFP,i} + p_2 D_{GFP,a} - d_r m_{GFP} \tag{12}$$

$$\frac{dm_{FLP}}{dt} = p_3 D_{FLP} - d_r m_{FLP} - d_c m_{FLP} shRNA \tag{13}$$

$$\frac{drtTA_a}{dt} = -f_3 U_{6,a} rtTA_a + r_3 U_{6,i} + f_4 DOX rtTA_i - r_4 rtTA_a \tag{14}$$

$$\frac{dshRNA}{dt} = p_4 U_{6,a} - d_r shRNA \tag{15}$$

Mapping to New Space

$$\frac{dZ}{dt} = t_l m_{FLP} - d_p P_{FLP} + 4 f_2 D_{GFP,c} \tag{16}$$

Reduced System in Z space

$$\frac{dP_{GFP}}{dt} = \frac{t_l p_1}{d_r} D_{GFP,i} + \frac{t_l p_2}{d_r} D_{GFP,a} - d_p P_{GFP} \tag{17}$$

$$\frac{dZ}{dt} = \frac{\frac{t_l p_3}{d_r} D_{FLP}}{1 + \frac{d_c}{d_r} shRNA} - d_p P_{FLP} + 4 f_2 D_{GFP,c} \tag{18}$$

$$\frac{dD_{GFP,a}}{dt} = f_2 D_{GFP,c} \tag{19}$$

$$D_{GFP,c} = D_{GFP,T} - D_{GFP,i} - D_{GFP,a} \tag{20}$$

$$D_{GFP,i} = \frac{(D_{GFP,T} - D_{GFP,a})}{1 + (\frac{P_{FLP}}{K_d})^4} \tag{21}$$

$$shRNA = \frac{\frac{p_4}{d_r} U_{6,T}}{1 + \frac{f_3}{f_1} rtTA_a} \tag{22}$$

Mapping back

$$\frac{dP_{FLP}}{dt} = \frac{part1 - part2}{1 + part3} \tag{23}$$

$$\begin{cases} part1 = \frac{\frac{t_l p_3}{d_r} D_{FLP}}{1 + \frac{d_c}{d_r} shRNA} - d_p P_{FLP} + 4 f_2 D_{GFP,c} \\ part2 = \frac{4 * f_2 D_{GFP,c}}{1 + \frac{f_1}{r_1} P_{FLP}^4} \\ part3 = \frac{16 \frac{f_1}{r_1} (D_{GFP,T} - D_{GFP,a}) P_{FLP}^3}{(1 + \frac{f_1}{r_1} P_{FLP}^4)^2} \end{cases} \tag{24}$$

Assuming No Retroactivity

$$\frac{dP_{GFP}}{dt} = \frac{t_l p_1}{d_r} D_{GFP,i} + \frac{t_l p_2}{d_r} D_{GFP,a} - d_p P_{GFP} \tag{25}$$

$$\frac{dP_{FLP}}{dt} = \frac{\frac{t_l p_3}{d_r} D_{FLP}}{1 + \frac{d_c}{d_r} shRNA} - d_p P_{FLP} + 4 f_2 D_{GFP,c} \tag{26}$$

$$\frac{dD_{GFP,a}}{dt} = f_2 D_{GFP,c} \tag{27}$$

$$D_{GFP,c} = D_{GFP,T} - D_{GFP,i} - D_{GFP,a} \tag{28}$$

$$D_{GFP,i} = \frac{(D_{GFP,T} - D_{GFP,a})}{1 + \frac{f_1}{r_1} P_{FLP}^4} \tag{29}$$

$$shRNA = \frac{\frac{p_4}{d_r} U_{6,T}}{1 + \frac{f_3}{f_1} rtTA_a} \tag{30}$$

Minimal ODE system

$$\frac{dP_{GFP}}{dt} = b_1 ngGFPRCN_{GFP,i} + b_2 ngGFPRNC_{GFP,a} - d_p P_{GFP} \tag{31}$$

$$\frac{dP_{FLP}}{dt} = \frac{b_3 RCN_{FLP}}{1 + \frac{b_4 RCN_{U6T}}{1 + b_5 RrtTA_a}} - d_p P_{FLP} + 4cRCN_{GFP,c} \tag{32}$$

$$\frac{dngGFPRCN_{GFP,a}}{dt} = F_2 ngGFPRCN_{GFP,c} \tag{33}$$

$$ngGFPRCN_{GFP,c} = ngGFPRCN_{GFP,T} - ngGFPRCN_{GFP,i} - ngGFPRCN_{GFP,a} \tag{34}$$

$$ngGFPRCN_{GFP,i} = \frac{(ngGFPRCN_{GFP,T} - ngGFPRCN_{GFP,a})}{1 + (\frac{P_{FLP}}{K_d})^4} \tag{35}$$

| Supplementary Note 4: Parameterization

Supplementary Note 4.1:: Fitting the Model

We used subsets of our data to parameterize our models. A summary of this process can be seen 5. As seen in equations 31 - 35 there is a subset of parameters that will be the same across all models reflecting the different

SUPPLEMENTARY TABLE 1 Chemical Reactions: Where D and P stand for plasmid DNA and proteins. Subscript "FLP" stands for the flippase, and "GFP" the green fluorescent protein. $D_{GFP,\,i}$ represents the GFP reporter plasmid with the terminator inhibiting transcription, and $D_{GFP,\,a}$ GFP reporter plasmid with the the terminator excised out. m and shRNA stand for mRNA and shRNA respectively. $rtTA_i$ represents $rtTA$ not bound to DOX; and $rtTA_a$ represent $rtTA$ bound to DOX. $U_{6,i}$ represent and repressed U_6 promoter and $U_{6,a}$ represents a uninhibited U_6 promoter.

Transcription	Degradation	Regulation
$D_{GFP,i} \xrightarrow{p1} D_{GFP,i} + m_{GFP}$	$m_{FLP} \xrightarrow{d_r} \varnothing$	$shRNA + mFLP \xrightarrow{d_c} shRNA$
$D_{GFP,a} \xrightarrow{p2} D_{GFP,a} + m_{GFP}$	$m_{GFP} \xrightarrow{d_r} \varnothing$	$4\,P_{FLP} + D_{GFP,i} \underset{r1}{\overset{f1}{\rightleftharpoons}} D_{GFP,c}$
$D_{FLP} \xrightarrow{p3} D_{FLP} + m_{FLP}$	$P_{FLP} \xrightarrow{d_p} \varnothing$	$D_{GFP,c} \xrightarrow{f2} 4\,P_{FLP} + D_{GFP,a}$
$U_{6,a} \xrightarrow{p4} U_{6,a} + shRNA$	$P_{GFP} \xrightarrow{d_p} \varnothing$	$rtTA_a + U_{6,a} \underset{r3}{\overset{f3}{\rightleftharpoons}} U_{6,i}$
Translation	$shRNA \xrightarrow{d_r} \varnothing$	***DOX Induction***
$m_{GFP} \xrightarrow{t_l} m_{GFP} + P_{GFP}$		$\varnothing \xrightarrow{p_r} rtTA_i$
$m_{FLP} \xrightarrow{t_l} m_{FLP} + P_{FLP}$		$rtTA_i + DOX \underset{r4}{\overset{f4}{\rightleftharpoons}} rtTA_a$
		$rtTA_i \xrightarrow{d_p} \varnothing$

topologies. These parameters are b_1, b_2, d_p, f_2 and c. On the other hand, K_d and components making up the b_f constant are expected to change for each Flp, shRNA level and circuit topology. Also b_f for the feedforward topology is also expected to change given different input levels. Therefore we constructed the following plan for fitting: We chose to fit d_p and b_2 to our maximal expression data described below. We chose to refit d_p and b_1 to the leaky expression data described below. Although these fits were not completely constrained to a single minima, looking at the error landscapes for these fits we were able to observe a linear relationship between optimal fits in both the leaky and maximal expression landscapes. Using the no shRNA dose response data, we incorporated these linear relationships when fitting the next set of parameters, b_2 (subsequently b_1 and d_p through the linear relationships), K_d, f_2, c and b_f for the no shRNA case. To fit b_f for the constitutive and feedforward systems we fixed all parameters except b_4 and b_5 and fit to the dose response data. To understand the effect of changing the ratio of TRE-Flp and U6-shRNA used in the system we fixed the parameters and simulated the system given different ratios of these plasmids.

Supplementary Note 4.2: The Linear Relationship between Fitting to Leaky and Max Data

As stated above we fit a subset of the parameters to the leaky expression and max expression data sets. The leaky expression data set was collected without the addition of recombinase to find the leaky expression of our reporter circuits, b_1. The max expression data set was collected using CAG expressed constitutive GFP as this will be very similar to the behavior of a cleaved reporter circuit. FACs data for both leaky and max time courses experiments were collected at 6, 12, 48 and 96 hours after transfection.

To fit b_1, b_2 and d_p to the leaky and max expression datasets we used a descent algorithm written in python to fit the data given different starting values for the parameters. In particular we fit d_p and b_1 using the leaky expression time course data set and d_p with b_2 for the maximal expression data set. We defined our target data to be the probability values of the distributions of the experimental data as they change over time. Our predicted data was then the simulated probability values for these same distributions over time. To optimized we minimized our loss function being the root mean squared error of the target and predicted distributions summed across time. A pictorial

SUPPLEMENTARY TABLE 2 List of all LMA derived ODE Species

Species	Definition
$D_{GFP,T}$	Total FRT-GRP reporter plasmids
$D_{GFP,i}$	# Inhibited FRT-GRP reporter plasmids
$D_{GFP,a}$	# Activated FRT-GRP reporter plasmids
$D_{GFP,c}$	# Complexes FRT-GRP reporter plasmids with Recombinase
D_{FLP}	The TRE-FLP plasmids
$U_{6,T}$	Total U_6 plasmids
$U_{6,i}$	# Inhibited U_6 plasmids
$U_{6,a}$	# Activated U_6 plasmids
P_{GFP}	GFP Protein
P_{FLP}	FLP Protein
$shRNA$	small hairpin RNA.
$rtTA_i$	inactive $rtTA$ Protein
$rtTA_a$	active $rtTA$ Protein (*i.e.* rtTA bound to DOX)
DOX	doxycycline
m_{FLP}	messenger RNA for FLP
m_{GFP}	messenger RNA for FLP

representation of these fits can be found in Fig. S16A and Fig. S17A. However many local minimum gave equivalent fits to these data. Looking at a sample of the error landscape we can see that as is often the case with a birth death process these parameters are linearly related (Fig. S16B,C and Fig. S17B,C). We fit these minima to estimate the linear relationship which we found to be $b_1 = 1 * d_p - 2$ for leaky expression and $d_p = 1 * b_2 - 2$ for maximal expression. For the maximal expression we found one local minima resided in a space where the system d_p was low enough to not effect the fit. As we believe d_p to be a governing reaction we did not include this point in the line fit for estimating the trend in the data, in effect setting a lower bound on d_p to be rate limiting. (Note: the representative fit was chosen using an initial delay that appeared to capture the data the best).To consider the error in the estimated the initial delay parameters, we repeated this process for two additional sets of initial delay parameters estimating the range of behavior seen with different initial delay's as measured in our color control experiments (*i.e.* μ_{ID} = 13.7, σ_{ID} 3.6 and μ_{ID} = 8.1, σ = 7.9). We found that the linear fits for one significant figure in both the slope and y-intercept are identical. Therefore we chose to move forward with $\mu_{\text{ID}} = 9.5$, $\sigma_{\text{ID}} = 5.5$ as the initial delay estimates. With this we then proceeded to use the linear relationships between these parameters to reduce the number of parameters we are fitting to the dose response data.

Supplementary Note 4.3: Constraining 5 Parameters to Fit the Dose Data

As expected constraining the remaining parameters presents a noteworthy challenge. To do this we started with the no-shRNA dose response data and first investigated the effect each parameter has on the corresponding fit. As b_2 is now related to d_p and b_1 via our fit linear relationships changing b_2 for our dose data does not affect the maximal

SUPPLEMENTARY TABLE 3 List of all LMA derived ODE Variables

Variable	Definition
p_1	m_{GFP} production rate from $D_{GFP,i}$
p_2	m_{GFP} production rate from $D_{GFP,a}$
p_3	m_{FLP} production rate from D_{FLP}
p_4	$shRNA$ production rate from $U_{6,a}$
t_l	translation rate
d_r	rate of RNA decay
d_p	rate of protein decay
d_c	rate for $shRNA$ mediated RISC complex degradation
f_1	binding rate of recombinase-DNA complex formation
r_1	unbinding rate of recombinase-DNA complex formation
f_2	rate of recombination and release of complex
f_3	binding rate of $rtTA_a$ with $U_{6,a}$ complex formation
r_3	unbinding rate of $rtTA_a$ with $U_{6,i}$ complex formation
f_4	binding rate of doxycycline to $rtTA_i$
r_4	unbinding rate of doxycycline to $rtTA_i$
p_r	zero order production rate of $rtTA_a$

The reaction involving d_c is taking in account that the RISC complexes with shRNA is recyclable [9].

level as much as the width of the states (*e.g.* a higher b_2 appears to decrease the width slightly). b_3 and K_d effect when we see the bimodal behavior appear in the dose response curve. f_2 sets the transition rate from the low to high mode. c for the parameter range we are in does not have a huge impact on the fit but does appear to affect the nonlinearity of transferring from the low to the high state for lower Flp levels. After our initial parameter sweep, we applied our descent algorithm to fitting these 5 parameters. Fitting over each DOX level in our dose response curve simultaneously by summing the loss function across each probability value over each dose. We then inspected the error traces in 1D for each parameter to get an idea of the position of any local minimum (Fig. S18 C). Each parameter is being constrained, in at least one dimension. Considering this we chose to move forward with the fitted values as recorded in table 5. Results of this fit are plotted in Fig. S19 and Fig. S20. Fig. S19 represents the fit to the full dose response data set. Fig. S20 is the result plot from only plotting the top 30 percent of the data using either the CFP or the simulated CFP value for determining the percentage. Next we fixed all parameters except b_4 and b_5 and repeated the process for the Constant and Feedforward dose response data to fit b_4 and b_5 respectively.

In order to be diligent we wanted to further explore relationships that may exist between b_f and K_d. To do this we fix all the other parameters and fit b_f (*i.e.* b_3, b_4 and b_5) and K_d to the corresponding dose response data given several initial conditions. When then plotted the error traces for these fits in the same manner as the leaky and maximal expression fits (Fig. 21). As discussed in a later supplemental note, non-dimensionalization of these equations also makes these linear relationships evident. Considering this we may find the non-dimensionalized space

SUPPLEMENTARY TABLE 4 List of all LMA derived ODE Species

Variable	Definition	Represents
b_1	slow birth rate of $D_{GFP,i}$	$\frac{rt_l p_1}{ngGFPd_r}$
b_2	fast birth rate of $D_{GFP,i}$	$\frac{rt_l p_2}{ngGFPd_r}$
b_3	production rate of P_{FLP}	$\frac{ngFLP\alpha t_l p_3}{d_r}$
b_4	Max $shRNA$ production	$\frac{ngshRNA\kappa p_4 d_c}{d_r^2}$
b_5	Scaled Effect of $rtTA_a$	$\frac{\eta f_3}{f_1}$
c	Scaled Cleavage and Dissociation Rate	$\frac{rf_2}{ngGFP}$
k_d	threshold for P_{FLP} .	$\sqrt[4]{\frac{r_1}{f_1}}$
F_2	Cleavage and Dissociation Rate	$\frac{f_2}{ngGFP}$
d_p	degradation rate of protein	d_p

useful in looking for trends in behavior and make predictions based on those trends.

To test our model, we calculated the predicted fold change overtime for all of the shRNA and Flp concentrations that were tested experimental. We then simulated each of these systems to compare the actual fold change with the predicted fold change. At 48hrs, the actual versus predicted plot gave us the expected 1:1 linear trend with an Pearson correlation value of around 0.9 for each system type (e.g. no-shRNA, constant-shRNA, feedforward-shRNA) Fig. 4.

SUPPLEMENTARY TABLE 5 Summary of Parameter Estimation

Parameter	Method	Value
d_p	Fit to Max and Leaky Data	$10^{1*log10(b_2)-2}(/hr)$
b_1	Fit to Leaky Data	$10^{1*log10(d_p)-2}(/hr)$
b_2	Fit to Max and no shRNA Dose Data	$10^{-0.26}(/hr)$
c	Fit to no shRNA Dose Data	$10^{-3.42}(/hr)$
f_2	Fit to no shRNA Dose Data	$10^{-1.57}(/hr)$
b_3	First Fit to no shRNA Dose Data	$10^{0.28}(/hr)$
K_d	First Fit to no shRNA Dose Dose Data	$10^{5.64}$
b_4	First Fit to Constant shRNA Dose Data	$10^{-4.5}(/hr)$
b_5	First Fit to Feedforward shRNA Dose Data	$10^{-7.25}(/hr)$

| Supplementary Note 5: Non-Dimensionalization and eKd

Supplementary Note 5.1: What do the non-dimensionalization terms represent?

Non-dimensionalization has been used in many engineering fields to explore the connection between physical parameters that govern key predictable features of the modeled system (*e.g.* Reynolds number). In our search to

describe how tolerant our system is to leaky expression we can non-dimensionalize the minimal model found in equations 31-35. This yields equations 36-40 with the non-dimensional parameters found in table 6. eB represent the ratio between the production rates of cleaved and uncleaved reporter TU driving GFP. eC includes many of the parameters that describe how recombinase molecules are recycled after they cleave their product. eF is the ratio of the formation of the cleaved reporter circuit over the degradation rate of proteins in the system. Finally, eKd is the ratio of the number of Flp molecules needed to cleave half the reporter circuits in the system with the parameters that control the production rate of Flp scaled by the degradation rate of all proteins in the system. As we are looking for a way to describe how tolerant the system is to leaky expression eKd appears to have grouped all the relevant parameters.

Supplementary Note 5.2: What does the tolerance to leaky expression (eKd) tell us about the tolerance to leaky expression?

When looking at the structure of our non-dimensionalized system specifically equation 40. We can see a relationship between $RCN_{GFP,i}$ and eKD that depend on P_{FLP}, where $RCN_{GFP,i}$ is the non-dimensionalized relative copy number of uncleaved reporter circuits and P_{FLP} is the non-dimensionalized level of P_{FLP} in the system. We can plot this relationship as seen in Fig. S22A. As P_{FLP} increase the number of $RCN_{GFP,i}$ goes down. Therefore eKd is the amount of P_{FLP} needed to lose half $RCN_{GFP,i}$. Changing parameters that increase the value of eKd, such as adding more shRNA to the system, increases the amount of P_{FLP} needed to convert half the $RCN_{GFP,i}$ to another form. It then follows that systems with higher eKd values are more tolerant to the leaky expression of P_{FLP}.

Supplementary Note 5.3: What is the relationship between non-dimensional eKd values and the physical ratio between Flp and shRNA?

eKd can also serve as a map for how the ratio between ng of Flp used in the system and ng of shRNA used in the system change this tolerance to leaky expression. Looking more deeply at the numbers grouped in the eKd terms we can see that to calculate eKd we need ng of Flp and shRNA used in the system and an approximation of the dox bound rtTA currently in the system. All of the other parameters in the eKd term are already approximated from fitting the data. ng for Flp and shRNA is pretty straightforward and we have chosen to approximate the dox bound rtTA used in the system by assuming it is linearly proportional to the amount of IFP expressed in the system. Fig. S22B illustrates how the tolerance changes given different amount of Flp and shRNA for the no-shRNA and constant topology. The apparent non-linear relationship can be seen in this 3D projection of this relationship where eKD is the z-axis(Fig. S22B). Fig. S22C,D demonstrate how changing rtTA bound to dox effects eKD. There are many insights we can begin to postulate from this relationship but to thoroughly explore even one of these insights is outside the scope of this paper. Considering this we will just leave you with some closing thoughts. First, given the non-monotonic trend of this non-linear relationship one should be able to achieve the same level of tolerance given different shRNA to Flp ratios. Second, identical tolerance to leaky expression should be achievable between the no, constant and feedforward topologies if shRNA and Flp are balanced to give the same eKd value. Altogether eKd, in theory, allows us to quantitatively compare tolerance to leaky expression for our digital enhancer topologies and identify trends in the data that should be useful for construction of optimally performing digital enhancers.

Non-Dimensional ODE system

$$\frac{dP_{GFP}}{dt} = RCN_{GFP,i} + eBRNC_{GFP,a} - P_{GFP} \tag{36}$$

$$\frac{dP_{FLP}}{dt} = RNC_{FLP} - P_{FLP} + eCRCN_{GFP,c} \tag{37}$$

$$\frac{dRCN_{GFP,a}}{dt} = eFRCN_{GFP,c} \tag{38}$$

$$RCN_{GFP,c} = RCN_{GFP,T} - RCN_{GFP,i} - RCN_{GFP,a} \tag{39}$$

$$RCN_{GFP,i} = \frac{(RCN_{GFP,T} - RCN_{GFP,a})}{1 + (\frac{P_{FLP}}{eK_d})^4} \tag{40}$$

SUPPLEMENTARY TABLE 6 Non-Dimensional Parameters

Variable	Represents
eB	$\frac{b_1}{b_2}$
eC	$4\frac{c^2}{b_f^2}$
eF	$\frac{F_2}{d_p}$
eK_d	$\frac{K_d d_p}{b_f}$
b_f	$\frac{b_3 RCN_{FLP}}{1+\frac{b_4 RCN_{U6T}}{1+b_5 RrtTA_a}}$

| Supplementary Note 6: InitialSynNotchResults

We test the efficacy of both our devices and our ability to predict their behavior by using them to enhance signal separation in a cell-to-cell communication system, synNotch. The synthetic notch system, synNotch, is an engineered version of the endogenous delta notch cell surface receptor, comprised of an external scFv (sensor) and internal effector domain (transcription factor) [10]. synNotch is highly modular and can be modified to respond to multiple membrane bound molecules by altering the external scFv portion of the receptor [10] [11] [12]. Upon binding its intended target, synNotch induces self-cleavage of the internal transcription factor to act upon a downstream target, such as a promoter of interest to induce the expression of a reporter gene. Here we chose to test a few of these systems and move forward with one of the higher performing and more widely used versions sensing CD19 and releasing tTA to activate a downstream target (Supplemental Fig. S27A). Previous characterization highlights the

power this system has for bioengineering applications from T cell reprogramming to tissue pattern formation [11] [13]. Little work, however, has been done to understand the composability of the synNotch system with downstream signal processing elements, which would aid in their incorporation into more complex cell circuitry. Here, we sought to compose synNotch together with our digital enhancers to show how cell-cell communication can be tuned in a predictable fashion.

To investigate the behavior of synNotch systems, we first transiently transfected three versions previously published by [10] into HEK293FT cells. We also varied the amount of synNotch receptor transfected into the system to study the concentration dependent behavior of each sensor (Supplemental Fig. S27). When cultured with CD19-expressing sender cells, we find that lower levels of transfected synNotch receptor improved performance for all three systems in receiver cells primarily because of the decrease in leaky output from uninduced receiver populations (Supplemental Fig. S27A). Due to the correlation between lower receptor level and a decrease in leaky expression, we posit that adding more receptors increases leaky expression through either accumulation of leaky cleavage and/or induced cleavage via membrane crowding. Increasing receptor level does not show any uniform, significant change in the overall output of induced cells when co-cultured with CD19 expressing sender cells, further supporting the use of minimal synNotch expression. To achieve sustainably low synNotch expression and better guarantee that all cells are expressing equal levels of the sensor we further explored the behavior of this system in a stably integrated context.

Both stable sender and stable receiver cells lines were built via lentivirus-mediated integration of membrane bound, hEF1a driven CD19 and hEF1a driven anti-CD19 synNotch-tTA, respectively. Downstream tre-tight promoters driving a fluorescent reporter, mKate2, were later transiently transfected into the receiver cells lines to complete the synNotch circuit, serving as a proxy for their sensing capacity. The leaky expression observed in this stably integrated system was significantly less than the transient system, suggesting that the receiver cells harbor few copy numbers of the synNotch sensor. These initial transient tests show cell signaling to generate 10 fold induction of the synNotch reporter (Supplemental Fig. S27B). However, we expect fold change to both increase and decrease based on how many receiver cells come into contact with sender cells. As such, we will use our digital enhancers to tune this output.

To test our digital enhancers with synNotch we took our stable cell line and transiently transfected in 3 shRNA to Flp ratios for both the constant and feedforward enhancer topology. In addition we tested an enhancer without the presence of shRNA, no-shRNA. We chose the ratios of shRNA to Flp to test the both the extreme behavior of the digitizer and also the models ability to predict this extreme behavior.

| Supplementary Note 7: SynBioHub Repository

Supplementary Note 7.1: Uploading Data to SynBioHub Introduction

We have chosen to upload our circuit data to SynBioHub. SynBioHub is an open-source project which allows scientists to store and share their synthetic biology design information [14]. Multiple instances of the SynBioHub repository have been established including the reference instance (`https://synbiohub.org`) and the NSF Living Computing Project (LCP) instance (`https://synbiohub.programmingbiology.org`) used by this work. The SynBioHub repository stores information using the Synthetic Biology Open Language (SBOL) [15], and it contains two different interfaces for various application scenarios: a web interface and the HTTP API. Anyone with a SynBioHub account can easily upload their DNA sequence and other design files, as well as experimental information to the SynBioHub repository. SynBioHub also incorporates a built-in visualization system using the SBOL Visual standard [16]. Once data is uploaded, it is placed in a private repository that can only be viewed by the user who uploaded it, as well as any individual provided a share link to this data (such as the reviewers for this paper). Once the data is ready for publication, it can be moved into a public repository, where it can be readily searched for using either the

web interface or HTTP API. For our digital enhancer we have uploaded both SBOL converted GenBank files for all constructs used in this paper, as well as spreadsheets describing how each construct was used. The corresponding FACs data collected for each experiment is also available upon request. The SynBioHub entry can be found on the LCP SynBioHub website (https://synbiohub.programmingbiology.org/public/DigitizingCommunication/DigitizingCommunication_collection/1). The DNA files contain the annotated DNA sequences of plasmids used in experiments. Each file's name is entitled using a unique ID followed by the annotated transcription unit, such as "BW2139(transcription unit)". The ID is used throughout experiments and recorded in the spreadsheets. The detailed description and the GenBank DNA files could be accessed and downloaded through the SynBioHub repository or the corresponding URLs on the spreadsheets.

The Excel spreadsheets record the details needed for experiment reproduction which have four tables in common:

- The "Experiment" table introduce necessary experiment information, such as the description of the experiments, the brief procedures of the experiment, the performer of the experiment and the explanation for the shortened key used in the "Sample" table;
- The "Sample" table gives information about the setup of the experiment groups as well as the names of the related data files;
- The "Experiment DNA sample" table contains the ID of the DNA sample used in the experimental group and the URLs linked to the corresponding DNA files on the SynBioHub.
- The "Calibration DNA sample" table contains the ID of the DNA used in the control group and the corresponding URLs.

Supplementary Note 7.2: Example Use of Database

The experiment-related files are organized in a way in which other researchers could use these files to reproduce the experiments and analyze the data quickly. For example, to repeat the specific experiment, one should open the corresponding experiment spreadsheet, such as "JHT6-LeakyExpressionData", go to the "Experiment DNA sample" or "Calibration DNA sample", click on the SynBioHub URLs and then downloads the DNA files. Once the researchers has the annotated DNA sequence, each plasmid can be synthesized de novo or modified from existing plasmids, and the DNA mixtures can be prepared with appropriate proportions noted on these two tables. After preparing the DNA mixtures well, one can follow the procedures on the "Experiment" table or the complete instructions of experiments on the "protocol" table. The FACs data referenced here can be converted to new standard, MEFL, to increase it comparability [17]. This can be accomplished with many available programs, we recommend TASBE tools (https://tasbe.github.io/) and Cytoflow (https://bpteague.github.io/cytoflow/). Once in MEFL scientist should be able to recreate any graph presented in the paper and even look at the data in new ways if so desired.

Supplementary Note 7.3: Why is this repository useful?

Using SynBioHub for experimental file collection promotes standardization and makes the experimental results more accessible for reproduction. Standardization is one of the most widely used concepts in the field of engineering. For synthetic biology, standardization means a series of consensus arrangements about experiment design, data collection and analysis, and information storage. With standardization, different parties could easily understand the goal of an experiment, the material, and the process needed to repeat the experiment. As an additional benefit, this should also aid in recapitulation of the methods applied to understand the experiments. There have been several trials of standardization in synthetic biology such as the iGEM (international genetically engineered machine) competition (https://igem.org/Main_Page), in which the contestants are requested to submit DNA sequences with annotations

to iGEM repository following the specific standards. We hope our use of this repository will also be seen as a trial of standardization encouraging others to reproduce our results. One reason for the current circumstance is the difficulty of finding a balance between flexibility and standardization: though the research methodology may be similar for synthetic biology, the experimental contexts and experimental procedures may vary a lot according to experiment goals and conditions. So designing the structure which can meet the various needs of researchers and compactly format the experiment information at the same time is challenging. However we believe we have arrived at a good balance as our structure was able to handle experiments from this paper as diverse as cell-cell communication to dose response curves. Another obstacle is lacking the reliable repository for DNA sequences and experiment information. GenBank and the journal's server may be the current option for researchers to upload their DNA and experiment details and is a great start; however we hope that more can be accomplished by also uploading our data to SynBioHub where we have a unified structure in data storage, which should encourage reproducibility.

| Supplementary Note 8: Toxicity

When examining the data for the doxycycline dose response curves, we note that as IFP expression increases with dox addition, CFP decreases (Fig. 26). There is a 2 fold decrease in CFP expression between the 0 nM and 225 nM dox concentrations tested, indicating an overall drop in protein production as more dox is added to the cells. Previous studies have suggested that dox can inhibit mammalian cell growth starting at concentrations as low as 200 nM, with significant toxicities observed in multiple mammalian cell lines with increasing drug addition [18] [19]. Our results seem to corroborate these findings; however, resource competition as mentioned in [8] may also provide an explanation. While it is not surprising that we observe decreased protein production due to dox, these findings may impact the reported values of fluorescent protein expression by falsely decreasing the overall output of each, and the subsequent model predictions of such outputs. This may provide a partial explanation for the unexpected shoulder in the off state populations when the hypothesized 'ideal' digital enhancer modules were composed with the synNotch sensors. In this configuration, the cells are no longer exposed to dox and thus may be producing a higher level of all proteins in the system.

| Additional Supplementary Tables

SUPPLEMENTARY TABLE 7 Modeling Actual Versus Predicted Results, pearson correlation, R, and absolute error, AE

Name	R (48hr)	AE (48hr)	R (96hr)	AE (96hr)
no-shRNA FC (n=16)	0.90	1.9	0.69	3.1
constant-shRNA FC (n=36)	0.95	1.8	0.85	3.4
forward-shRNA FC (n=36)	0.97	1.7	0.88	3.2
no-shRNA AUC (n=16)	0.79	1.2	0.52	1.3
constant-shRNA AUC(n=36)	0.96	1.1	0.88	1.2
forward-shRNA AUC(n=36)	0.93	1.1	0.82	1.1
no-shRNA SNR (n=16)	0.75	2.1	0.42	1.7
constant-shRNA SNR (n=36)	0.69	1.9	0.64	2.4
Forward0shRNA SNR (n=36)	0.64	3.1	0.65	2.1
no-shRNA SNR >-10 (n=14)	0.85	1.9	0.40	1.6
constant-shRNA SNR >-10 (n=25)	0.35	1.6	0.02	2.4
Forward0shRNA SNR >-10 (n=28)	0.75	3.0	0.47	2.1

SUPPLEMENTARY TABLE 8 Modeling Actual Versus Predicted Results for Cell-Cell Communication Experiment

	Input			Output			Model		
**ng Flp to ng shRNA Ratio**	**FC**	**AUC**	**SNR**	**FC**	**AUC**	**SNR**	**FC**	**AUC**	**SNR**
35:5 Constant-shRNA	8.33	0.82	-3.82	13.77	0.80	-0.99	16.61	0.81	-1.31
35:1 Constant-shRNA	7.22	0.80	-6.88	1.32	0.54	-7.88	3.50	0.70	-3.35
1:5 Constant-shRNA	9.90	0.83	-3.98	1.93	0.63	-5.51	2.48	0.66	-5.04
15:20 Feedforward-shRNA	7.81	0.81	-4.04	10.46	0.78	-1.19	11.55	0.76	-1.51
1:20 Feedforward-shRNA	9.33	0.83	-6.47	3.40	0.68	-2.51	3.08	0.64	-4.37
15:1 Feedforward-shRNA	8.79	0.81	-3.83	3.44	0.68	-5.48	11.12	0.80	-2.51
1:0 no-shRNA	10.42	0.83	-4.29	8.16	0.75	-1.79	20.96	0.81	-1.19
1:5 Feedforward-shRNA	8.67	0.82	-6.88	4.79	0.71	-2.22	5.53	0.70	-4.00

Additional Supplementary Figures

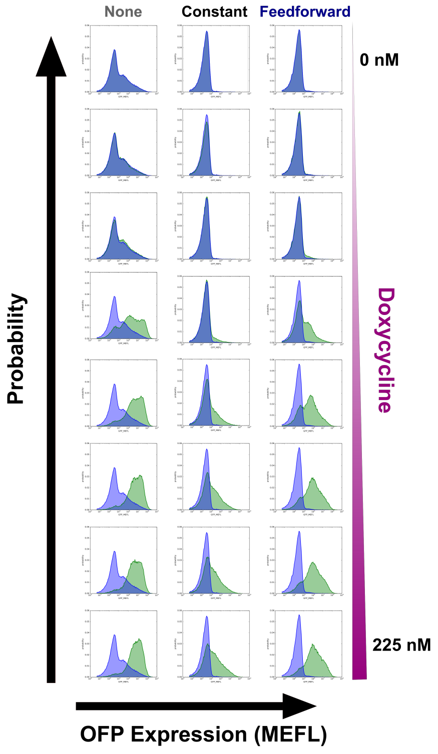

SUPPLEMENTARY FIGURE 1 Dose response histograms show different trends between digital enhancer topologies Doxycycline dose response data from all three digital enhancer topologies 48 hours after transient transfection into HEK293FT cells with 1:10 Flp:shRNA plasmid ratios for the constant and feedforward topologies. Data represents all events compiled from three technical replicates (n = 3) collected via flow cytometry 48 hours post transfection of HEK293FT cells

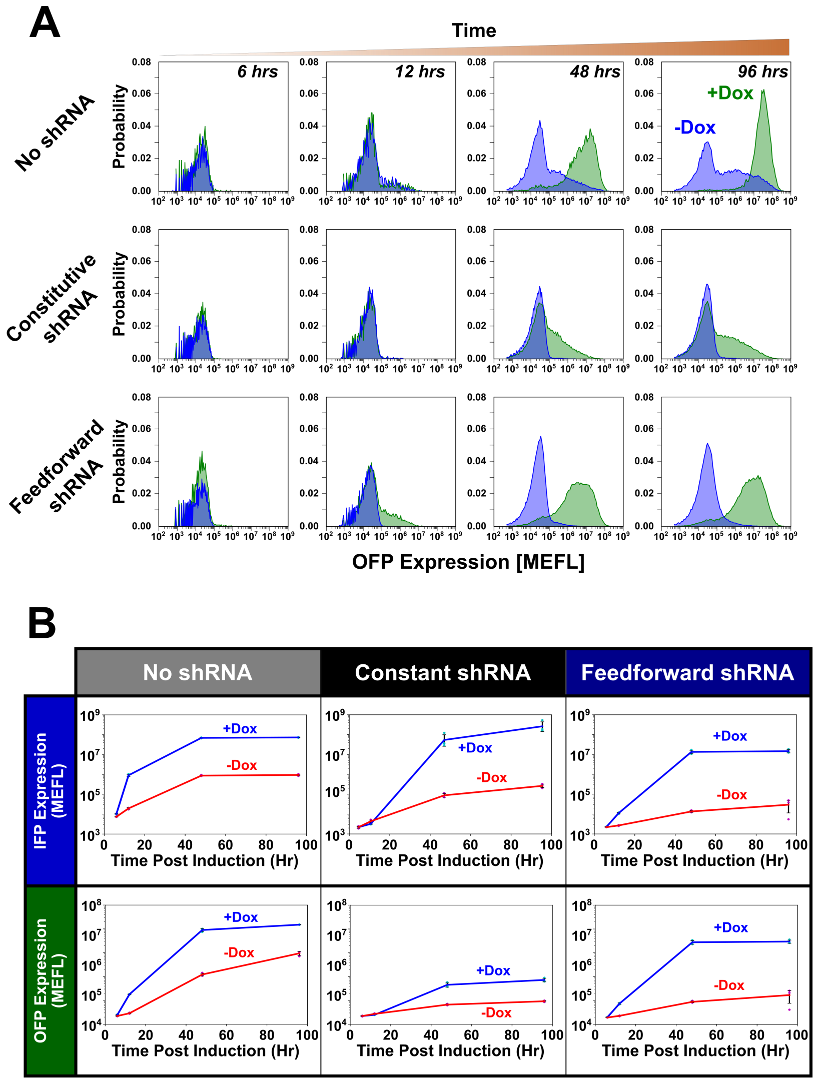

SUPPLEMENTARY FIGURE 2 A) Histogram data up to 96 hours for the first iteration of each digital enhancer topology displays unique population dynamics. B) Geometric mean data for IFP (top) and OFP (bottom) up to 96 hours post transient transfection in HEK293FT cells for the no shRNA (left), constant shRNA (middle), and feedforward shRNA (right) digital enhancer topologies at a Flp:shRNA ratio of 1:10. All data in A represent one of three technical replicates (n = 3) of HEK293FT cells transfected at t = 0, and data in B represent the SEM of the technical replicates for each time point and dox condition.

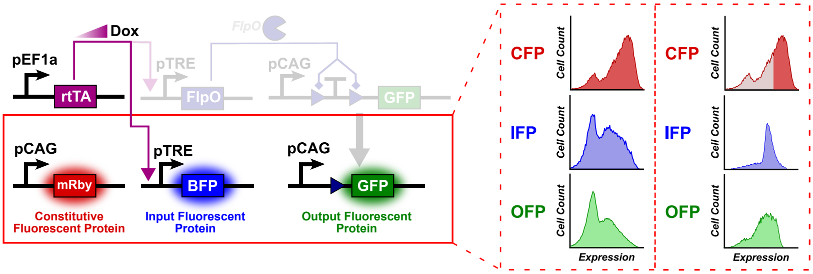

SUPPLEMENTARY FIGURE 3 Fluorescent protein proxy system allows dissection of circuit components Fluorescent reporters were incorporated into each transfection mix to report on different aspects of the digital enhancer components. A constitutive mRuby plasmid (Constitutive Fluorescent Protein, CFP) was included to give an idea of relative plasmid plasmid copy numbers per cell through quantitative assessment of marker fluorescence via flow cytometry. An rtTA responsive BFP (pTRE-BFP) was included to report on the activity of the TRE promoter driving the Flp recombinase (Input Fluorescent Protein, IFP), giving an idea of how much Flp transcript is produced at different levels of doxycycline addition. Finally, a plasmid containing a CAG promoter and GFP sequence sandwiching a transcription termination sequence flanked by frt sites reports on the activity of Flp protein (Output Fluorescent Protein, OFP). This system is shown at left, and the right panels demonstrate an example of how gating for different populations of CFP expressing cells can highlight different trends in the data. Populations analyzed are denoted by the dark red shading of the CFP histogram in each box, with IFP and OFP histograms representative of populations after CFP gating.

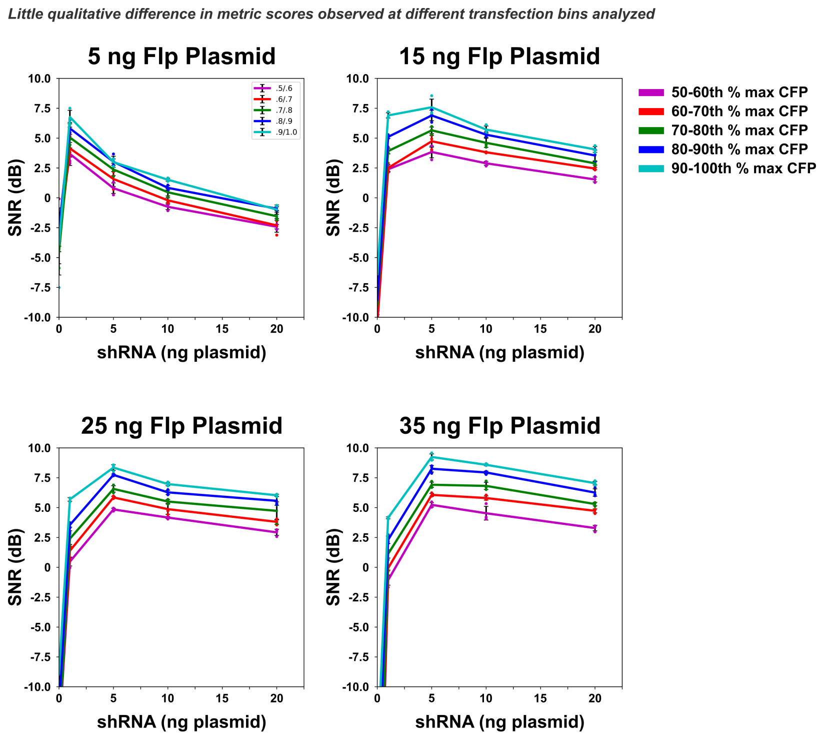

SUPPLEMENTARY FIGURE 4 OFP SNR values plotted by transfection bin at 48 hrs post induction of representative constitutive-shRNA digitizer designs, where darker colors indicate higher transfection bins. Within each individual Flp:shRNA ratio, while the quantitative values of SNR vary between transfection bins, the qualitative trend, with the exception of one outlier, is the same for all bins. Data points represent the average of three technical replicates (n=3) of transfected HEK293FT cells, error bars represent the SEM.

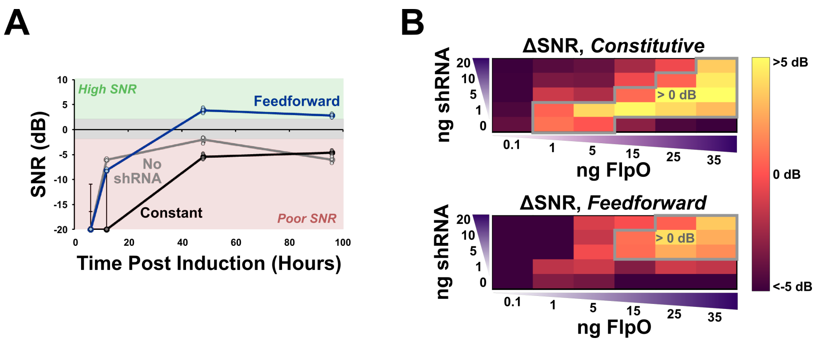

SUPPLEMENTARY FIGURE 5 Varying Flp:shRNA ratios affects digital enhancer performance (A) Output SNR calculations over time for each digital enhancer topology. Each line represents 5 ng of a pTRE-Flp plasmid transfected with 50 ng of the corresponding shRNA plasmid. Heat maps in (B) represent ΔSNR values calculated at 48 hours post transient transfection of each topology, highlighting variations in the performance of each at different Flp:shRNA plasmid weight ratios in HEK293FT cells (n = 3). Error bars represent the SEM of technical replicates. Note: error bars are expected to be large for low (-20 dB) SNR values due to the large effect of noise at this level.

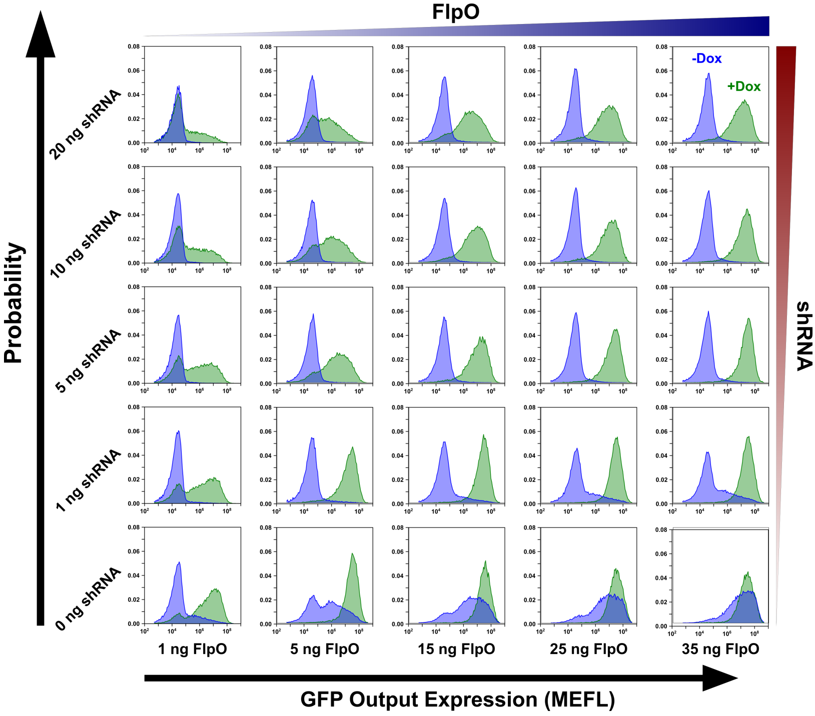

SUPPLEMENTARY FIGURE 6 Constant shRNA digital enhancer histogram matrix Histogram matrix of all Flp:shRNA ratios tested for the constant shRNA topology. Histograms represents combined flow cytometry data of three technical replicates (n = 3) collected 96 hours post transfection of digital enhancers in HEK293FT cells

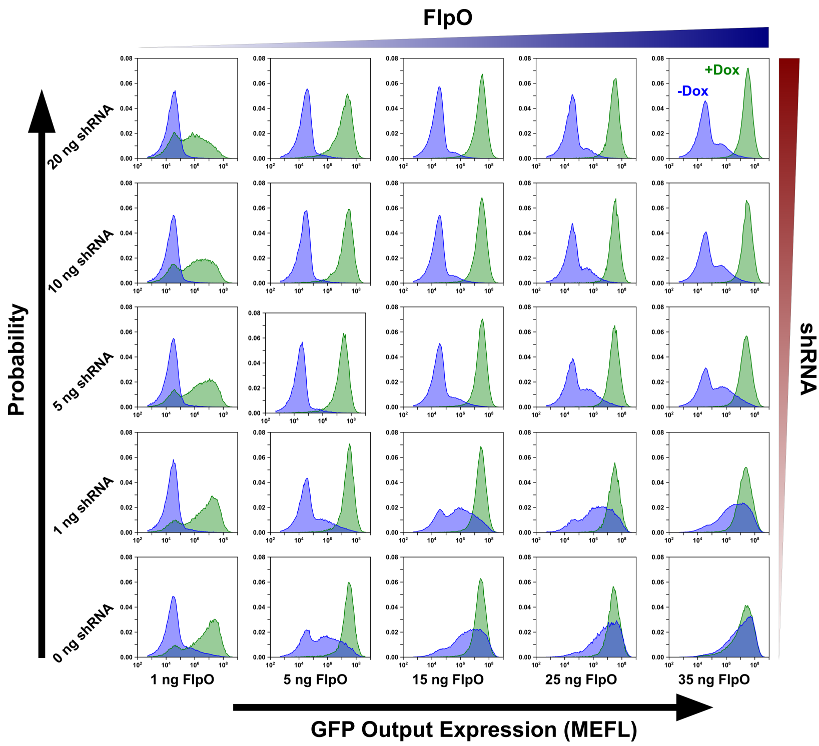

SUPPLEMENTARY FIGURE 7 Feedforward shRNA digital enhancer histogram matrix Histogram matrix of all Flp:shRNA ratios tested for the feedforward shRNA topology. Histograms represents combined flow cytometry data of three technical replicates (n = 3) collected 96 hours post transfection of digital enhancers in HEK293FT cells

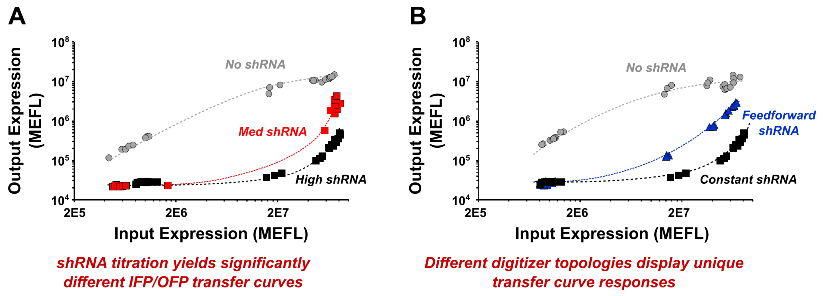

SUPPLEMENTARY FIGURE 8 shRNA titration modulates the transfer curve of each digital enhancer topology independent of input strength (A) Constant shRNA regulated topologies transfected with Flp:shRNA ng weight ratios of 5:0 (gray line), 5:5 (red line), and 5:20 (black line) display high variability in OFP expression despite similar levels of IFP expression, indicating an ability to tune digital enhancer performance in response to variable input levels in HEK293FT cells 48 post transient transfection (n = 3). (B) No, constant, and feedforward shRNA topologies exhibit differing responses to the same level of input after 48 hours post transient transfection into HEK293FT cells despite the constant and feedforward topologies containing the same level of transfected shRNA-expressing plasmid (n = 3). *Note: Lines are drawn to highlight trend, not fit to data.*

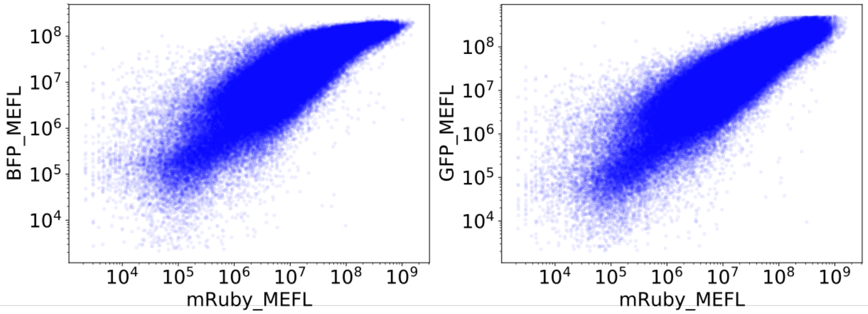

SUPPLEMENTARY FIGURE 9 Linear Trend in Color Control: Scatter plot of 3D color control data. The left panel is the project on the mRuby MEFL, BFP MEFL dimension. The right panel is the projected data onto the mRuby MEFL, GFP MEFL dimension.

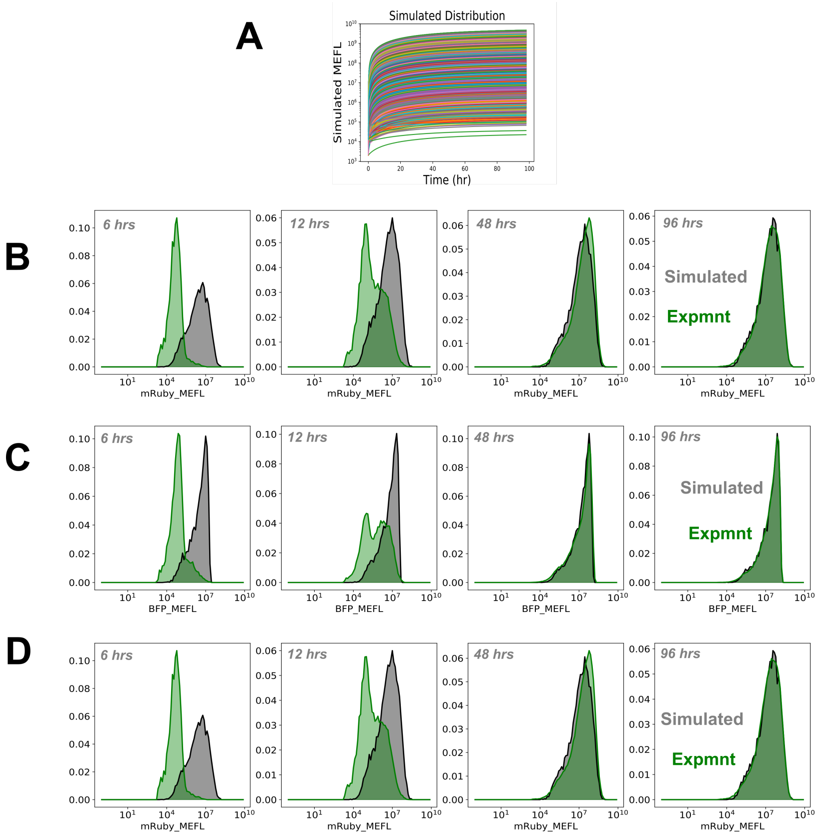

SUPPLEMENTARY FIGURE 10 No Cell Division or Initial Delay: Panel A is a time course plot where each individual cell simulated representative fluorescent value is plotted in a different color. Panel B-D are time course histograms depicting the cell population respective fluorescence values over time. Green histograms represent physical experimental data where the grey histograms represent simulated data.

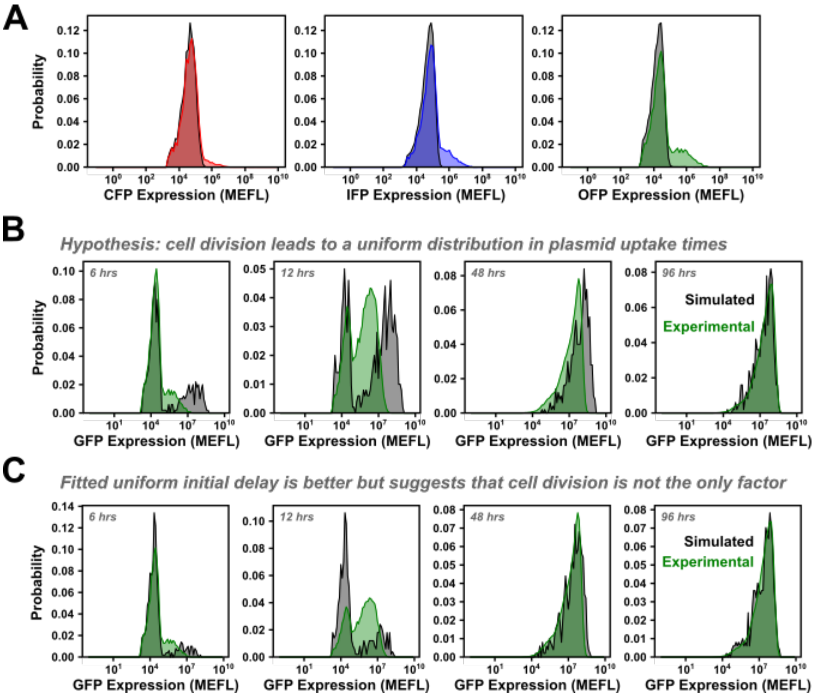

SUPPLEMENTARY FIGURE 11 Cell Division and Uniform Initial Delay: Panel A is the Overlaid probability distributions of the wild type 96 hour time point plotted in grey and multi sample 6 hour time point fluorescence data plotted in corresponding color for the fluorophore used, green, blue and red. In Panel B the histograms for cell division time of 19.8 before the fit and 50 after the fit. Left to right the histograms represent the 6hr, 12hr, 48hr and 96hr respectively. Panel C is of the histograms after adding in a uniform initial delay. Left to right the histograms represent the 6hr, 12hr, 48hr and 96hr respectively. Green distributions are experimental data and black distributions are the simulated data for the CAG GFP sample.

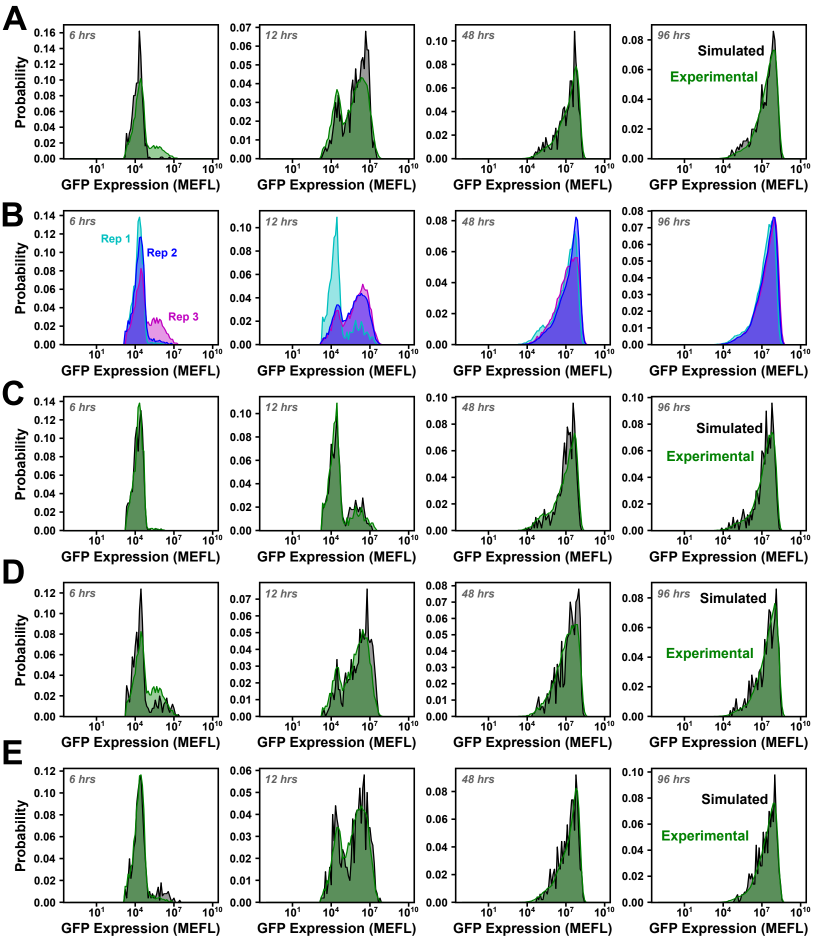

SUPPLEMENTARY FIGURE 12 A Gaussian Distributed Initial Delay: Panel A is the time course plot with fit gaussian initial delay. The histograms left to right are 6hr, 12hr, 48hr and 96hr respectively. Green distributions are experimental data and black distributions are the simulated data for the CAG GFP sample. Panel B is probability distributions of the Multi-Color control with the Biological replicates plotted in different colors. Panels C-E are the biological replicates fit individually in the same manner.

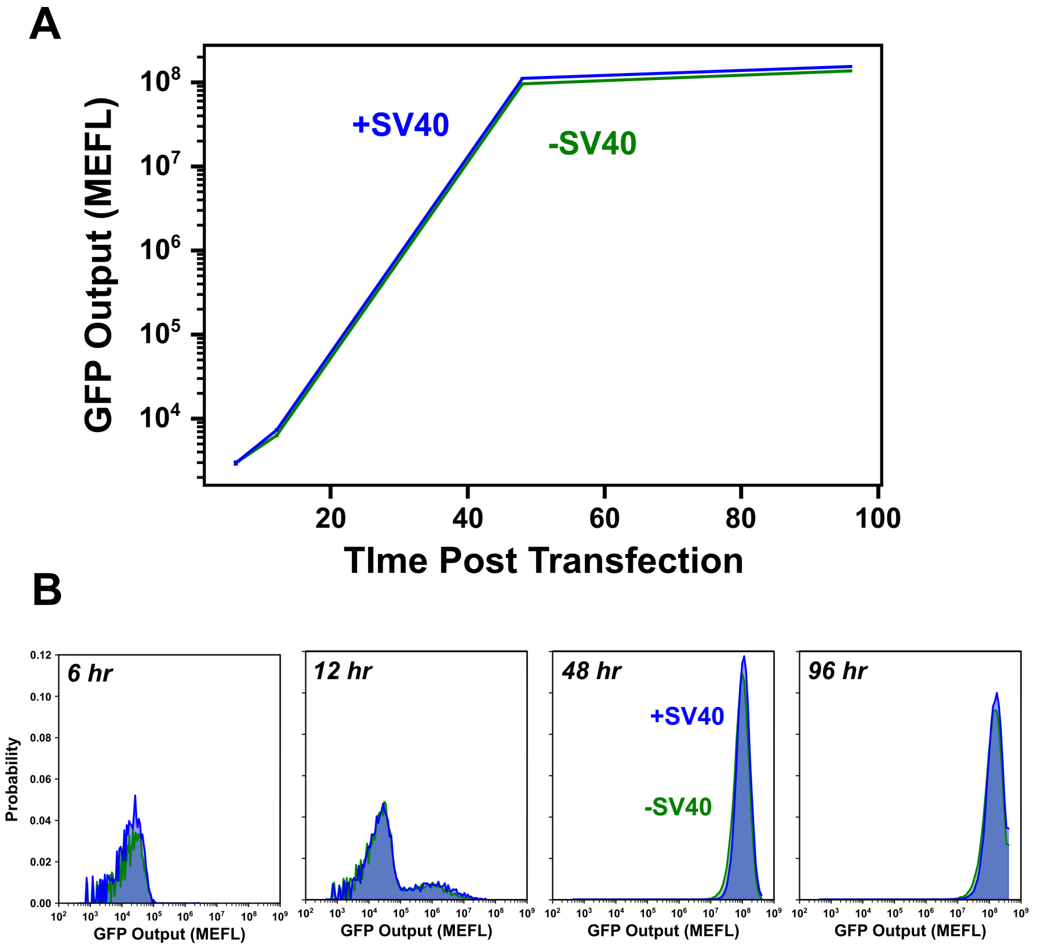

SUPPLEMENTARY FIGURE 13 Plasmid Dilution: Panel A is the geometric mean GFP expression up to 96-hours post transfection from CAG-GFP plasmids containing or lacking the SV40 ORI (+SV40/-SV40) in HEK293FT cells. Panel B contains histogram plots of population data contributing to the plot in A. Data points represent the average geometric mean of three technical replicates (n=3) of transfected HEK293FT cells, error bars represent the SEM.

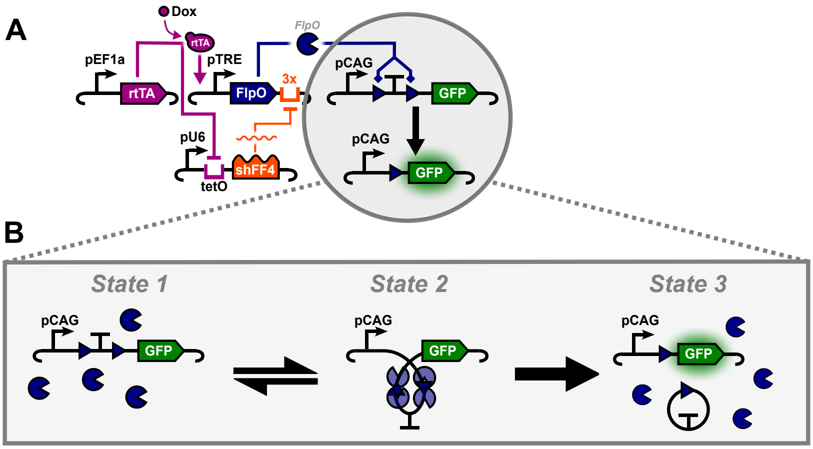

SUPPLEMENTARY FIGURE 14 Recombinase Reaction States: Panel A represents a condensed diagram in which all states of the recombination reaction are represented as a single process. Panel B provides a breakdown of the hypothesized states of the reaction. State 1 depicts the uncleaved reporter circuit after rtTA has induced Flp recombinase production, and State 2 shows a system in which both frt sites are fully occupied by Flp. The transition between States 2 and 3 represents the formation of a Holliday junction between the two frt sites and Flp proteins, where State 3 shows the intended cleaved reporter in the 'on' configuration, where GFP is constitutively expressed from the CAG promoter and the transcriptional terminator has been removed from the reporter.

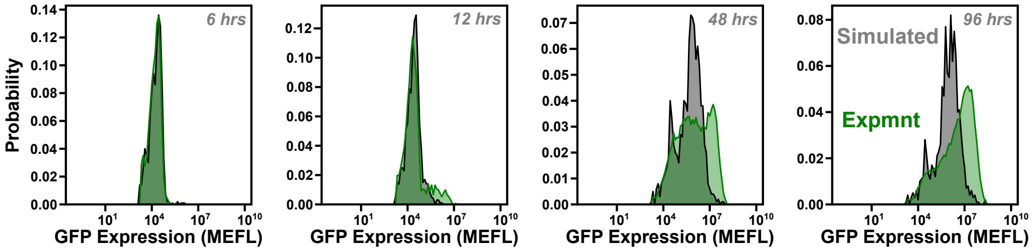

SUPPLEMENTARY FIGURE 15 Time Course of Sequestration Effect: A representative time course showing the repressive effect in the distribution due to the added state.

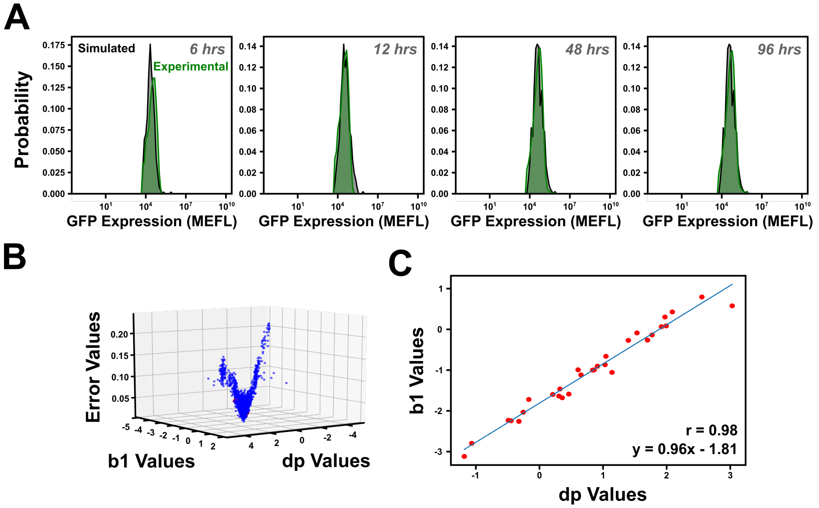

SUPPLEMENTARY FIGURE 16 Leaky Fit: Panel A is a representative image of the time course fit to the leaky expression data, 6hr, 12hr, 48hr and 96hr respectively. Green distributions are experimental data and black distributions are the simulated data for the CAG terminator GFP sample. Panel B is a sample of the fit error landscape. Blue dots represent points along the error trace sampled during the decent and red points represent local minima arrived given different initial conditions. Panel C is a linear fit indicating the relationship between the local minima given different initial delay parameters.

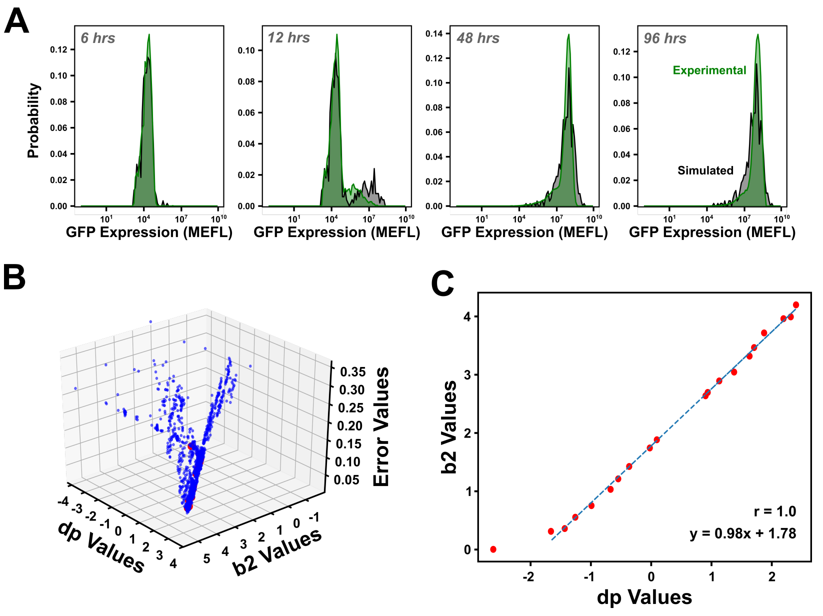

SUPPLEMENTARY FIGURE 17 Max Fit: Panel A is a representative image of the time course fit to the leaky expression data, 6hr, 12hr, 48hr and 96hr respectively. Green distributions are experimental data and black distributions are the simulated data for the CAG GFP sample. Panel B is a sample of the fit error landscape. Blue dots represent points along the error trace sampled during the decent and red points represent local minima arrived given different initial conditions. Panel C is a linear fit (excluding minima where b2 has little impact on minima) indicating the relationship between the local minima given different initial delay parameters.

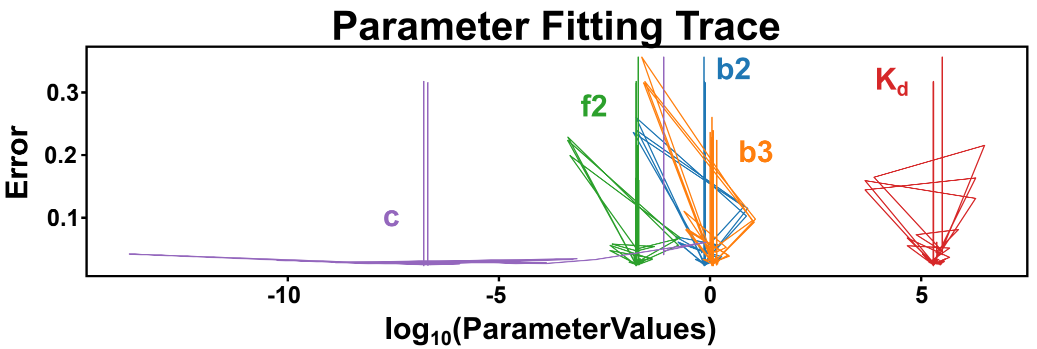

SUPPLEMENTARY FIGURE 18 Constrained Fit: A one dimensional projection of each parameter in this 5 parameter fit where the x-axis is the value of the indicated parameter and the y axis is the squared error of the error trace.

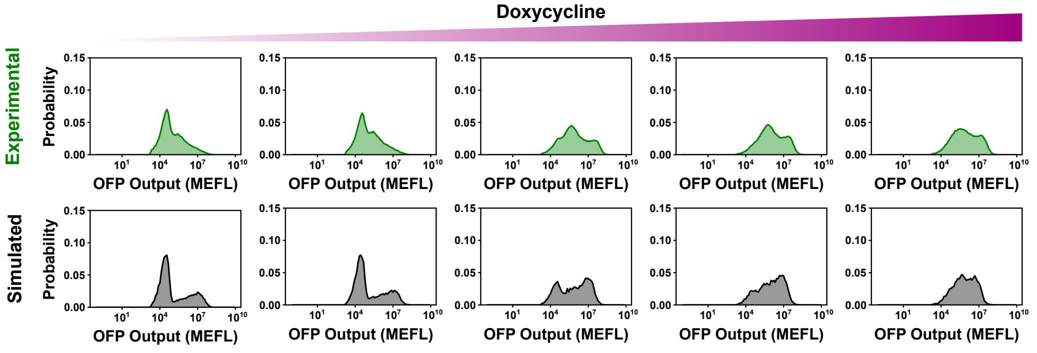

SUPPLEMENTARY FIGURE 19 Full Dose Fit: Each Panel represent a different DOX concentration. The green probability distribution is the experimental data and the black distribution is the simulated probability distribution

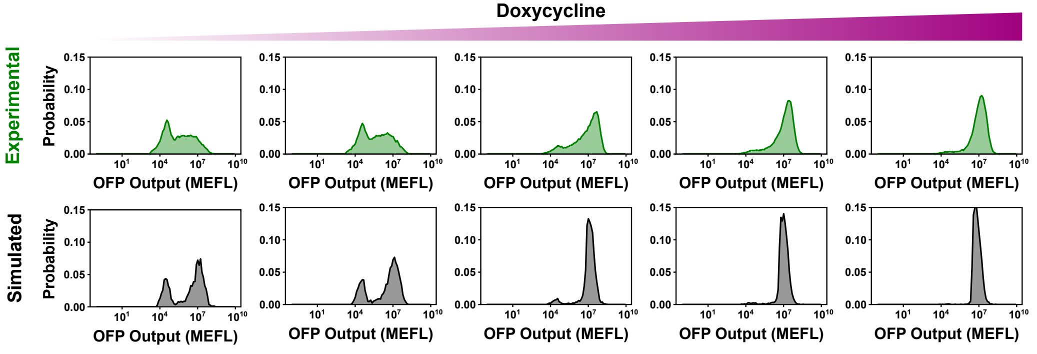

SUPPLEMENTARY FIGURE 20 Dose Fit: Each Panel represent a different DOX concentration. The green probability distribution is the experimental data for the top 30% of transfected cells as determined by the CFP distribution. The black distribution is the simulated probability distribution for the top 30% of simulated transfected cells as determined by the simulated CFP distribution.

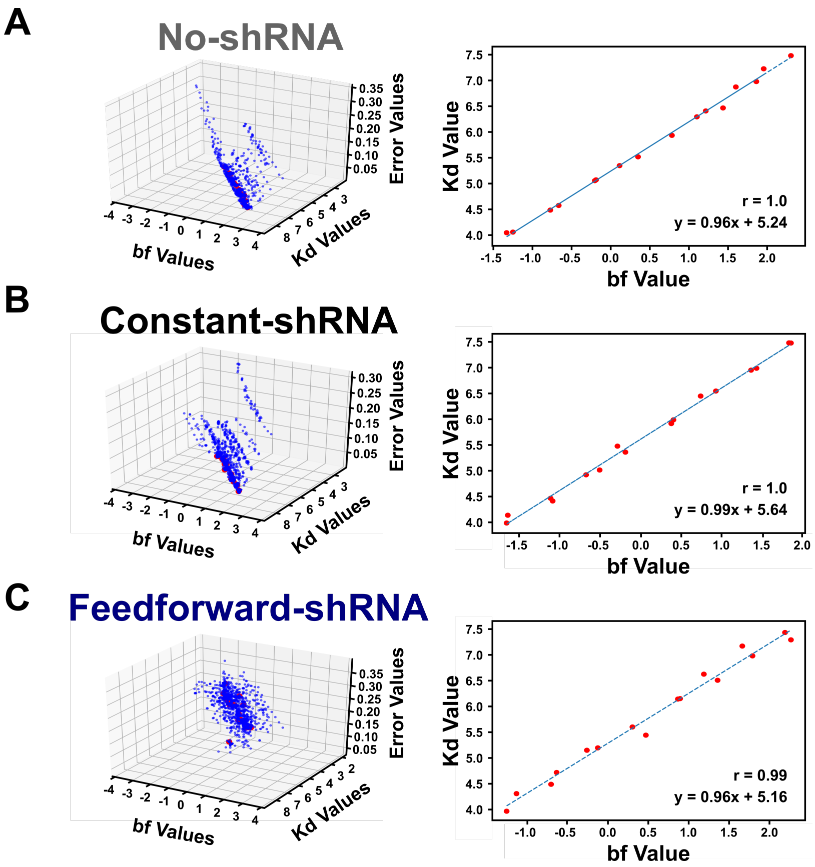

SUPPLEMENTARY FIGURE 21 bf versus Kd Fit Landscape: Samples of the fit error landscape. Blue dots represent points along the error trace sampled during the decent and red points represent local minima arrived given different initial conditions. The linear fit indicates the relationship between the local minima given different initial conditions. Panel A is for the no-shRNA bf and Kd values. Panel B is for the constant-shRNA and panel C is for the feedforward-shRNA case.

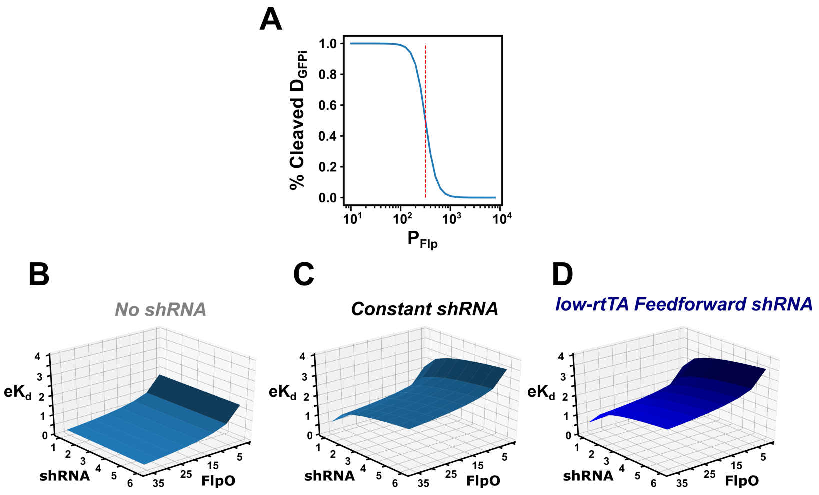

SUPPLEMENTARY FIGURE 22 eKd: Panel A Is a line plot depicting how the percentage of cleaved promoters changes as P_{FLP} increases. eKd is marked on this plot in red and will increase or decrease the amount of P_{FLP} needed to cleave half the reporter circuit for half of the cells in a population. Panel B represents how eKd changes with respect to how much ng of Flp or shRNA is used in the system for the constant shRNA expression topology. Panels C and D represent how eKd changes for the feedforward topology given different amounts of $rtTA_a$ created at different DOX levels.

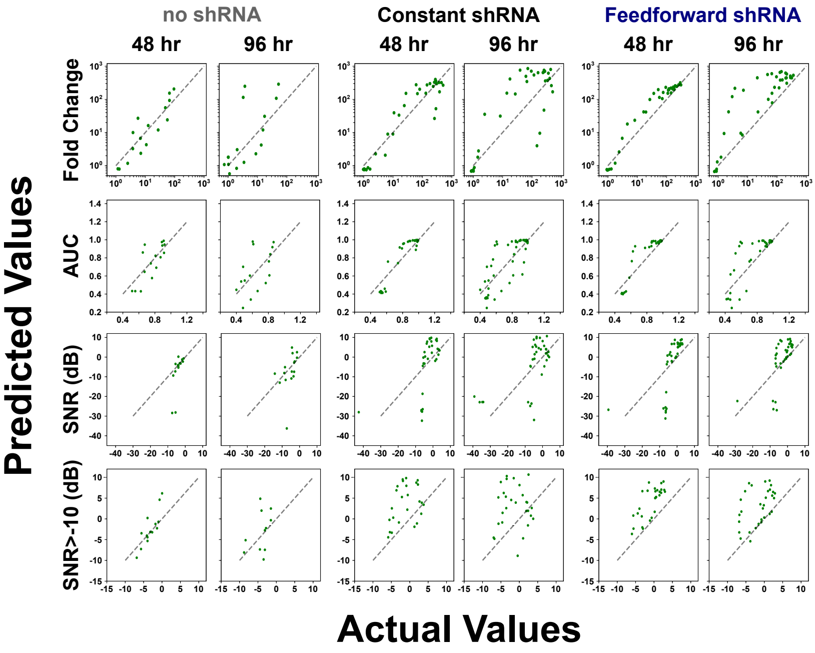

SUPPLEMENTARY FIGURE 23 Actual versus predicted plots for 48hr and 96hr time points in all three topologies across all three metrics. The last row shows SNR actual versus predicted values excluding SNR values less than -10.

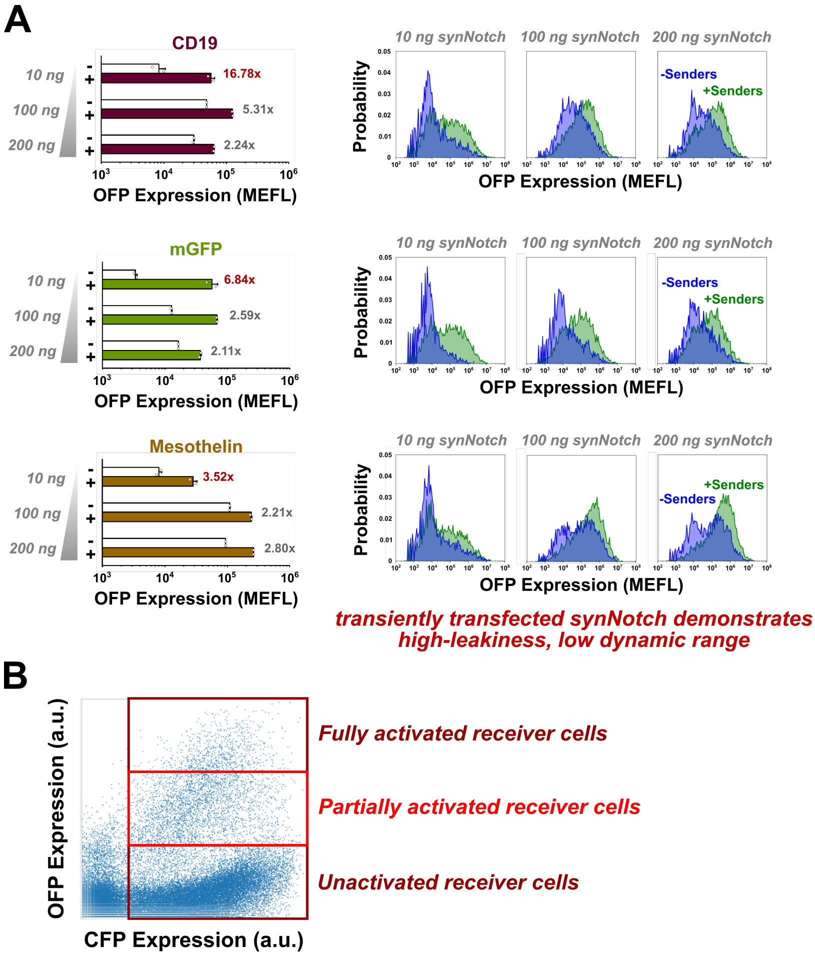

SUPPLEMENTARY FIGURE 24 Transient synNotch tests show weak dynamic range (A) CD19 (top), GFP (middle), and Mesothelin (bottom) synNotch receptors display weak dynamic ranges across several levels of transfection (left) and poor separability between cell states (right). Bar chart data represents the average geometric mean of three technical replicates (n=3) of transfected HEK293FT cells, error bars indicate the SEM. (B) Integrated CD19 synNotch receiver HEK293FT cells activated to varying degrees when co-cultured with CD19-expressing sender HEK293FT cells over a 48-hr period. Partially activated receivers are the best candidates for implementation of the digital enhancers to ensure they are fully activated when mixed with sender cells.

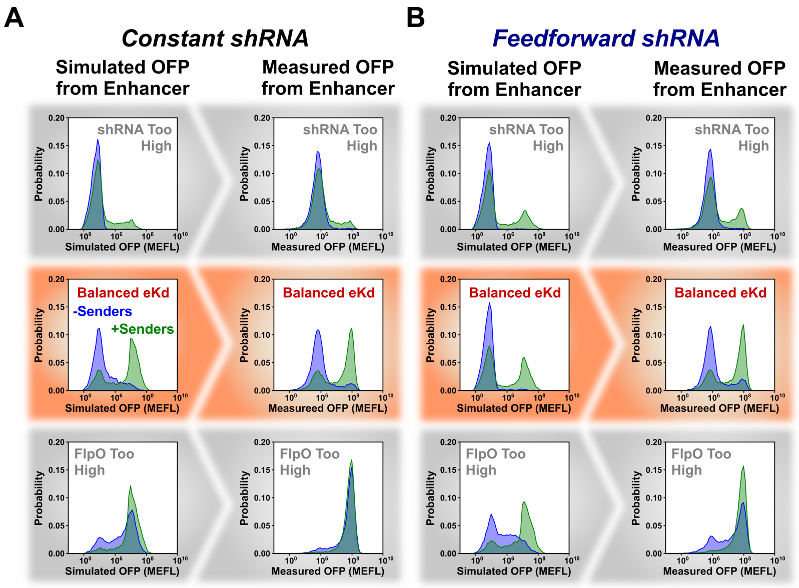

SUPPLEMENTARY FIGURE 25 synNotch-digital enhancer modelling predicts qualitative response of several digital enhancer topologies Modelling predictions (left) vs experimentally derived (right) HEK293FT populations expressing the constant (A) or feedforward (B) digital enhancer topologies. Three conditions are considered for each topology, in which eKd is hypothesized to be too high (top, i.e. cells unable to switch on), optimal (middle, i.e. low leaky basal activity while cells are permitted to switch on when the synNotch receptor is activated), and low (bottom, i.e. cells exhibit a large amount of basal leaky OFP expression).

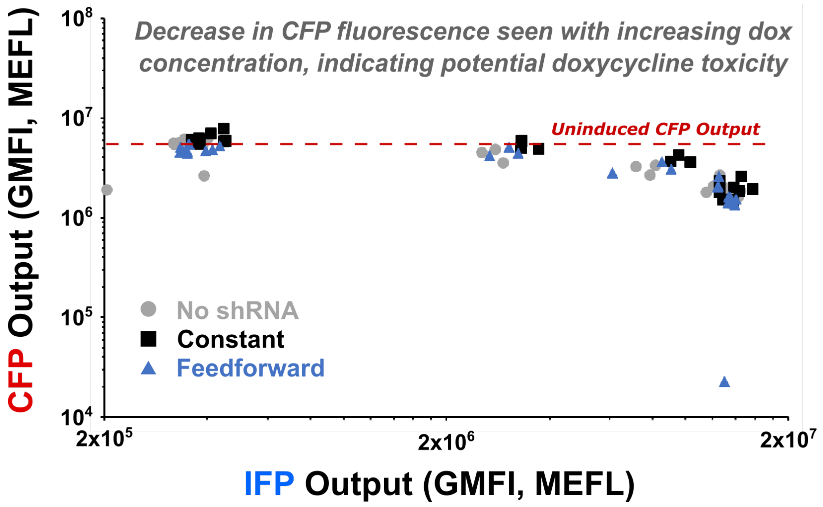

SUPPLEMENTARY FIGURE 26 Doxycycline-associated toxicity seen as drop in CFP fluorescence A plot of CFP vs IFP indicates decreased levels of CFP expression with increasing levels of IFP express (*i.e.* increasing dox levels). This may indicate doxycycline-associated effects on the cell which cause protein production levels to drop. Data points represente the average geometric mean of three technical replicates (n=3) of transfected HEK293FT cells, error bars indicate the SEM.

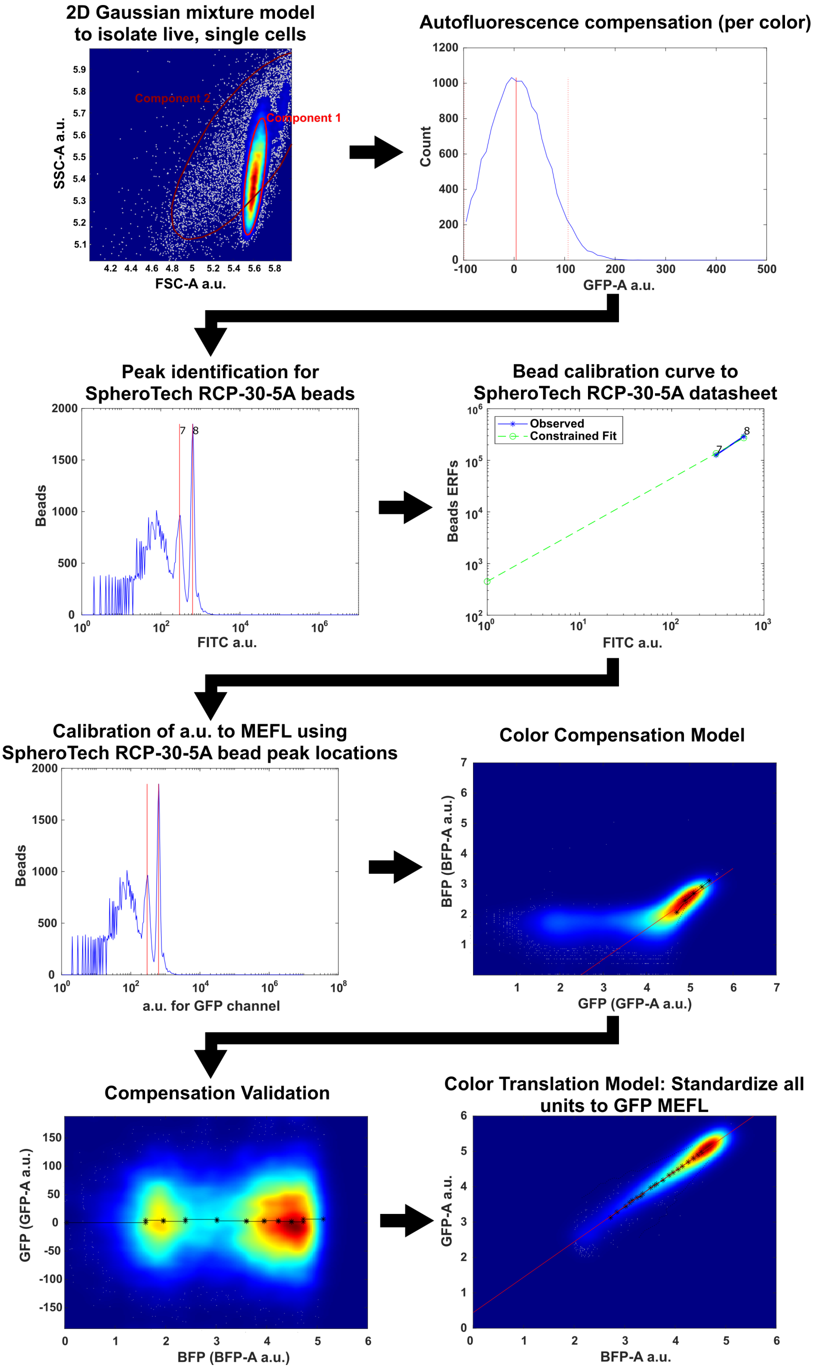

SUPPLEMENTARY FIGURE 27 A representative workflow for processing flow cytometry data collected for all experiments shown in this manuscript. Populations of cells are first gated for live, single cells using a 2D Guassian mixture model and autofluorescnece correction for each fluorescent protein collected on each filter is performed using HEK293FT cells transfected with a null plasmid. All fluorescence data collected is then standardized to a single unit of Molecules of Equivalent Fluorescein (MEFL) using data from SpheroTech RCP-30-5A beads collected on each fluorescence channel. Compensation is performed to determine and correct the level of bleedthrough of each fluorophore into unintended channels, and finally all fluorescence data is standardized to GFP MEFL using a sample containing all fluorescent proteins driven by the same promoter (from different plasmids) transfected in equal amounts.

references

[1] Davidsohn N, Beal J, Kiani S, Adler A, Yaman F, Li Y, et al. Accurate Predictions of Genetic Circuit Behavior from Part Characterization and Modular Composition. ACS Synth Biol 2015;4:673–681.

[2] Wang J, Isaacson S, Belta C. Modeling Genetic Circuit Behavior in Transiently Transfected Mammalian Cells. ACS Synth Biol 2019;8:697–707.

[3] Wang J, Isaacson S, Belta C. Predictions of Genetic Circuit Behaviors Based on Modular Composition in Transiently Transfected Mammalian Cells. In: 2018 IEEE Life Sciences Conference (LSC); 2018. p. 85–88.

[4] Lillacci G, Benenson Y, Khammash M. Synthetic control systems for high performance gene expression in mammalian cells. Nucleic Acids Research 2018;46:9855–9863.

[5] Milo R, Phillips R, Cell Biology By the Numbers; last viewed June, 2019. `http://book.bionumbers.org/`.

[6] Kredel S, Oswald F, Nienhaus K, Deuschle K, Rocker C, Wolff M, et al. mRuby and a Bright Monomeric Red Fluorescent Protein for Labeling of Subcellular Structures. Plos One 2009;.

[7] Subach O, Cranfill P, Davidson M, Verkhusha V. An Enhanced Monomeric Blue Fluorescent Protein with the High Chemical Stability of the Chromophore. Plos One 2011;6.

[8] Qian Y, Huang H, Jimenez J, Vecchio D. Resource Competition Shapes the Response of Genetic Circuits. ACS Synth Biol 2017;6:1263–1272.

[9] Dana H, Chalbatani G, Mahmoodzadeh H, Karimloo R, Rezaiean O, Moradzadeh A, et al. Molecular Mechanisms and Biological Functions of siRNA. Int J Biomed Sci 2017;13:48–57.

[10] Morsut L, Roybal K, Xiong X, Gordley R, SCoyle, Thomson M, et al. Engineering Customized Cell Sensing and Response Behaviors Using Synthetic Notch Receptors. Cell 2016;164:780–791.

[11] Roybal K, Rupp L, Morsut L, Walker W, McNally K, Park J, et al. Precision Tumor Recognition by T Cells With Combinatorial Antigen-Sensing Circuits. Cell 2016;164:770–779.

[12] Cho J, Okuma A, Al-Rubaye D, Intisar E, Junghans R, Wong W. Engineering Axl specific CAR and SynNotch receptor for cancer therapy. Sci Rep 2018;8.

[13] Toda S, Blauch L, Tang S, Morsut L, Lim W. Programming self-organizing multicellular structures with synthetic cell-cell signaling. Science 2018;361:156–162.

[14] McLaughlin J, Myers C, Zundel Z, Mısırlı G, Zhang M, Ofiteru I, et al. SynBioHub: A Standards-Enabled Design Repository for Synthetic Biology. ACS Synthetic Biology 2018;7:682–688.

[15] Roehner N, Beal J, Clancy K, Bartley B, Misirli G, Grünberg R, et al. Sharing Structure and Function in Biological Design with SBOL 2.0. ACS Synthetic Biology 2016;5:498–506.

[16] Beal J, Nguyen T, Gorochowski T, Goñi-Moreno A, Scott-Brown J, McLaughlin JA, et al. Communicating Structure and Function in Synthetic Biology Diagrams. ACS Synthetic Biology 2019;8:1818–1825.

[17] Beal J, weiss R, Yaman F, Davidsohn N, Adler A. A Method for Fast, High-Precision Characterization of Synthetic Biology Devices. Computer Science and Artificial Intelligence Laboratory Technical Report 2012;.

[18] Ermak G, Cancasci V, Davies K. Cytotoxic effect of doxycycline and its implications for tet-on gene expression systems. Analytical Biochemistry 2003;318:152–154.

[19] Sourdeval M, Lemaire C, Brenner C, Boisvieux-Ulrich E, Marano F. Mechanisms of doxycycline-induced cytotoxicity on human bronchial epithelial cells. Frontiers in Bioscience 2006;11:3036–3048.
