# Supplementary material for: Quantitative characterization of recombinase-based digitizer circuits enables predictable amplification of biological signals
Source: Commun Biol. 2021 Jul 15;4:875. doi: 10.1038/s42003-021-02325-5 (PMC8282836; doi:10.1038/s42003-021-02325-5)
Supplement: Supplementary file 3 — Reporting Summary [file 42003_2021_2325_MOESM3_ESM.pdf]

nature research

Corresponding author(s): Ron Weiss, Wilson Wong and Jake Beal

Last updated by author(s): Nov 20, 2020

Reporting Summary

Nature Research wishes to improve the reproducibility of the work that we publish. This form provides structure for consistency and transparency in reporting. For further information on Nature Research policies, see our Editorial Policies and the Editorial Policy Checklist.

Statistics

For all statistical analyses, confirm that the following items are present in the figure legend, table legend, main text, or Methods section.

n/a	Confirmed	
☐	☒	The exact sample size (*n*) for each experimental group/condition, given as a discrete number and unit of measurement
☐	☒	A statement on whether measurements were taken from distinct samples or whether the same sample was measured repeatedly
☐	☒	The statistical test(s) used AND whether they are one- or two-sided<br>*Only common tests should be described solely by name; describe more complex techniques in the Methods section.*
☒	☐	A description of all covariates tested
☐	☒	A description of any assumptions or corrections, such as tests of normality and adjustment for multiple comparisons
☐	☒	A full description of the statistical parameters including central tendency (e.g. means) or other basic estimates (e.g. regression coefficient) AND variation (e.g. standard deviation) or associated estimates of uncertainty (e.g. confidence intervals)
☒	☐	For null hypothesis testing, the test statistic (e.g. *F*, *t*, *r*) with confidence intervals, effect sizes, degrees of freedom and *P* value noted<br>*Give P values as exact values whenever suitable.*
☒	☐	For Bayesian analysis, information on the choice of priors and Markov chain Monte Carlo settings
☒	☐	For hierarchical and complex designs, identification of the appropriate level for tests and full reporting of outcomes
☐	☒	Estimates of effect sizes (e.g. Cohen's *d*, Pearson's *r*), indicating how they were calculated

Our web collection on statistics for biologists contains articles on many of the points above.

Software and code

Policy information about availability of computer code

Data collection	All simulations were done using custom code via Python 3.7. All images were assembled using Inkscape. All Flow Cytometry Data was processed with TASBE Flow Analytics package available through the open source software development platform Github (https://github.com/TASBE/TASBEFlowAnalytics). For each experiment, the standard set of controls detailed in Data Collection was used to convert all raw fluorescence data to a standard Molecules of Equivalent Fluorescein (MEFL) unit.
Data analysis	All metric calculations were done using either the SciPy module or custom code written in Python 3.7.

For manuscripts utilizing custom algorithms or software that are central to the research but not yet described in published literature, software must be made available to editors and reviewers. We strongly encourage code deposition in a community repository (e.g. GitHub). See the Nature Research guidelines for submitting code & software for further information.

Data

Policy information about availability of data

All manuscripts must include a data availability statement. This statement should provide the following information, where applicable:
- Accession codes, unique identifiers, or web links for publicly available datasets
- A list of figures that have associated raw data
- A description of any restrictions on data availability

Data for all figures and results presented here, as well as plasmid maps for all plasmids used in this study, is available for download from the SynBioHub repository at: https://synbiohub.programmingbiology.org/public/DigitizingCommunication/DigitizingCommunication_collection/1

Field-specific reporting

Please select the one below that is the best fit for your research. If you are not sure, read the appropriate sections before making your selection.

☒ Life sciences ☐ Behavioural & social sciences ☐ Ecological, evolutionary & environmental sciences

For a reference copy of the document with all sections, see nature.com/documents/nr-reporting-summary-flat.pdf

Life sciences study design

All studies must disclose on these points even when the disclosure is negative.

Sample size	See Flow Cytometry Section
Data exclusions	To compare across cells with similar plasmid copy number a transfection marker was incorporated in the study. Analysis was done comparing cells found in the top 30% of transfection marker expression. For a subset of the data multiple transfection bins were used; and as presented in the supplemental even at low transfection the results are qualitatively the same.
Replication	All attempts at replication where successful. The most striking evidence of replication success can be seen in the ability to characterize the digitizer systems at Boston University, BU, and test the usability of this characterization by composing the digitizers built at BU with a synNotch system constructed at Massachusetts Institute of Technology by different researchers in different labs.
Randomization	See Flow Cytometry Section
Blinding	See Flow Cytometry Section

Reporting for specific materials, systems and methods

We require information from authors about some types of materials, experimental systems and methods used in many studies. Here, indicate whether each material, system or method listed is relevant to your study. If you are not sure if a list item applies to your research, read the appropriate section before selecting a response.

Materials & experimental systems

n/a	Involved in the study	
☒	☐	Antibodies
☐	☒	Eukaryotic cell lines
☒	☐	Palaeontology and archaeology
☒	☐	Animals and other organisms
☒	☐	Human research participants
☒	☐	Clinical data
☒	☐	Dual use research of concern

Methods

n/a	Involved in the study	
☒	☐	ChIP-seq
☐	☒	Flow cytometry
☒	☐	MRI-based neuroimaging

Eukaryotic cell lines

Policy information about cell lines

Cell line source(s)	HEK293FT cellline purchased from ATCC
Authentication	none
Mycoplasma contamination	cells not tested
Commonly misidentified lines (See ICLAC register)	NA

Flow Cytometry

Plots

Confirm that:

- [x] The axis labels state the marker and fluorochrome used (e.g. CD4-FITC).
- [x] The axis scales are clearly visible. Include numbers along axes only for bottom left plot of group (a 'group' is an analysis of identical markers).
- [x] All plots are contour plots with outliers or pseudocolor plots.
- [x] A numerical value for number of cells or percentage (with statistics) is provided.

Methodology

Sample preparation	For digitizer performance transiently-transfected HEK293FT cells in 48w plates were detached from the plate using 50 uL of 0.05% Trypsin-EDTA solution and resuspended using 75 uL of DMEM containing 5% FBS, 1% L-glutamine, 1% penicillin/ streptomycin, and 1% sodium pyruvate. Cells were thoroughly mixed by pipetting to dislodge cells from the plate and ensure a single-cell suspension for flow cytometry. Samples were transfered to a 96w plate and 85/125 uL of each sample was run on the Attune NxT flow cytometer using an autosampler attachment.<br><br>For synNotch experiments a comparable sample prep protocol was followed.
Instrument	All data for experiments characterizing digitizer performance were collected using an Attune NxT flow cytometer with an attached Autosampler (Life Technologies). The Attune was equipped with violet (405 nm), blue (488 nm), yellow (561 nm) and red (605 nm) excitation lasers and the default filter configuration for each (http://tools.thermofisher.com/content/sfs/ manuals/100024236_AttuneNxT_SW_UG.pdf).<br><br>Experimental data for the synNotch sensor module, both transient and integrated, as well as the composition of the sensor and digitizer modules were collected using the BD LSRFortessa flow cytometer. This instrument was equipped to measure eGFP and eYFP (488 nm laser, 530 \slash 30 emission filter), mtagBFP2 (405 nm laser, 450/50 emission filter), mKate (561 nm laser, 610 \slash 20 emission filter), iRFP720 (640 nm laser, 780\slash 60 emission filter). Cells were trypsinized, resuspended and run on the Fortessa in a similar manner as described using the Attune.
Software	The use of fluorescent proteins as a proxy for component performance within our devices requires that the production of these proteins be quantitatively comparable. To ensure this, we make use of the TASBE Flow Analytics package available through the open source software development platform Github (https://github.com/TASBE/TASBEFlowAnalytics). For each experiment, the standard set of controls detailed in Data Collection was used to convert all raw fluorescence data to a standard Molecules of Equivalent Fluorescein (MEFL) unit. Data was further processed using custom Python scripts to isolate populations of cells based on expression patterns.
Cell population abundance	Cells are gated to isolate live, single cells expressing a transfection marker. All samples display >89% viability on the live gate, and live/singlet/transfected cell populations analyzed are >5,000 events (i.e. cells) per sample.
Gating strategy	Live cells are gated for based on size exclusion from an FSC/SSC plot, and single cells are further isolated by gating cells falling on the diagonal line of an FSC-A vs FSC-H plot. All transfected cells contain a constitutively expressed fluorescent protein that is used as the transfection marker. Live/single cells are further gated for expression of this marker and are broken into transfection bins based on the marker's relative expression level in each cell.

- [x] Tick this box to confirm that a figure exemplifying the gating strategy is provided in the Supplementary Information.
